# Patterns of *Aedes aegypti* immature ecology and arboviral epidemic risks in peri-urban and intra-urban villages of Cocody-Bingerville, Côte d’Ivoire: Insights from a dengue outbreak

**DOI:** 10.1371/journal.pone.0324893

**Published:** 2026-04-30

**Authors:** Yasmine N. Biré, Julien Z. B. Zahouli, Jean D. K. Dibo, Pierre N. Coulibaly, Prince G. Manouana, Jacques F. Mavoungou, Fanny Hellhammer, Gaël D. Maganga, Luc S. Djogbenou, Ayola A. Adegnika, Steffen Borrmann, Stefanie C. Becker, Mahama Touré

**Affiliations:** 1 Centre d’Entomologie Médicale et Vétérinaire, Université Alassane Ouattara, Bouaké, Côte d’Ivoire; 2 Centre Suisse de Recherches Scientifiques en Côte d’Ivoire, Abidjan, Côte d’Ivoire; 3 Centre de Recherches Médicales de Lambaréné, Lambaréné, Gabon; 4 Institute for Tropical Medicine, University of Tübingen, Tübingen, Germany; 5 Institut de Recherche en Ecologie Tropicale, Centre National de la Recherche Scientifique et Technologique, Libreville, Gabon; 6 Working Group for Vector Associated Biodiversity and Infection, University of Veterinary Medicine Hanover, Hanover, Germany; 7 Research Center for Emerging Infection and Zoonoses, University of Veterinary Medicine Hanover, Hanover, Germany; 8 Centre Interdisciplinaire de Recherches Médicales de Franceville, Franceville, Gabon; 9 Tropical Infectious Diseases Research Centre, University of Abomey-Calavi, Cotonou, Benin; 10 German Center for Infection Research, Tübingen, Germany; CEA, FRANCE

## Abstract

**Background:**

Cocody-Bingerville, southeastern Côte d’Ivoire, a traditional focus of yellow fever (YF), has faced outbreaks of dengue (DEN) that caused 4,371 cases and 29 deaths from 2023–2024. However, local *Aedes* vector studies and arboviral outbreak responses have mostly focused on urban neighborhoods including intra-urban villages, but no prior research has been done in peripheral villages. We compared *Aedes aegypti* indices, container productivity, and DEN and YF epidemic risks between peri-urban and intra-urban villages during the outbreaks.

**Methods:**

From August 2023 to July 2024, we sampled *Aedes* eggs, larvae and pupae among three peri-urban and three intra-urban villages. Sampling was done in domestic and peridomestic ecozones of 100 households in each village per survey, and uniformly across four climatic seasons: short dry, short rainy, long dry, and long rainy seasons. We compared *Ae. aegypti* container productivity, *Stegomyia* indices (house index: HI, container index: CI, and Breteau index: BI) and pupal indices (pupae per house index: PHI, pupae per container index: PCI, and pupae per person index: PPI) across villages, ecozones and seasons.

**Results:**

*Aedes aegypti* widely dominated *Aedes* fauna in both peri-urban (98.1%) and intra-urban (99.8%) villages. The most productive containers were small containers (31.1%), tires (30.5%) and medium containers (20.1%) in the peri-urban villages, and tires (64.6%) and small containers (18.7%) in the intra-urban villages that yielded over 80% of all the pupae collected in each village type. These key containers produced substantially more pupae in the domestic ecozones (70.9%) in the peri-urban villages, but equitably between the domestic (48.8%) and peridomestic (51.2%) ecozones in the intra-urban villages. In all villages, key containers provided over 80% of pupae sampled during short dry and long rainy seasons. CI, HI and BI were comparable between the peri-urban (29.9%, 35.9% and 41.4) and intra-urban (36.7%, 48.0% and 56.2) villages. However, PCI (3.38 *vs*. 1.26 pupae/container), PHI (5.18 *vs.* 1.75 pupae/house) and PPI (1.25 *vs*. 0.54 pupae/person) values were, respectively, 2.7, 3.0 and 2.3-fold higher in the intra-urban compared to peri-urban villages. Lower pupal indices in the intra-urban villages were compensated by five additional *Aedes* vector species. All indices were correlated to rainfall in all villages, with correlation coefficients varying from 0.16 to 0.84.

**Conclusion:**

In Cocody-Bingerville, all sampled peri-urban and intra-urban villages hosted high densities of *Ae. aegypti* immatures and habitats (tires, small or medium containers). *Stegomyia* indices remained consistently high, exceeding WHO DEN and YF epidemic thresholds in all villages, potentially contributing to ongoing DEN outbreaks. *Aedes* vector surveillance and outbreak responses should be extended to peri-urban villages, as they are likely contributors to arbovirus persistence and reintroduction. This is the first study to directly compare *Aedes* indices across peri-urban and intra-urban settings during an arboviral outbreak and offers a baseline for strategically reducing human exposure. Community-led interventions (larval source reduction, larviciding, public awareness) targeting identified larval habitats could help control arboviral outbreaks.

## Introduction

*Aedes* mosquito-borne arboviruses – including dengue virus (DENV), yellow fever virus (YFV), chikungunya virus (CHIKV) and Zika virus (ZIKV) – pose a growing global public health threat, particularly in Africa where over 831 million people (approx. 70% of population) are at risk [1–4]. In 2023 alone, 171,991 suspected cases of dengue (DEN), including 70,223 cases and 753 deaths were reported from 15 African countries, including Côte d’Ivoire [3]. In Africa, arboviruses are mostly transmitted to humans by *Aedes aegypti* and *Aedes albopictus* species [1]. The spread and geographical expansion of these two invasive vector species and arboviruses are mainly driven by urbanization and climate change [1,2].

Rapid and uncontrolled urbanization in Africa provides the creation of millions of water-holding containers and the accumulation of solid or plastic waste items (e.g., tires, cans,) that can serve as suitable ovipositing and larval breeding sites for *Aedes* mosquitoes, all which increase vector population density and the risk of arbovirus transmission to humans [4,5]. Additionally, an increased density and size of human population in urbanized areas offer large blood-feeding sources for *Aedes* females [4,6]. Containers are filled with water manually by people (domestic items) or naturally by rainfall (outdoor or discarded items) making them suitable for the larval development of *Ae. aegypti* through all year round [4,5]. Human behaviors related to water and container use are often associated with local climate, and these interactions promote *Ae. aegypti* populations [7]. Indeed, rainwater wet larval breeding sites leading to *Aedes* egg-laying and hatching, while high temperature and high relative humidity (RH) can accelerate larval development and increase adult survivorship [8,9]. Climatic conditions therefore intensifies the frequency of human bites, resulting in an increased risk of transmission and shift of disease burden from malaria to arboviruses in Africa [1,6,10,11]. However, in Africa, the vaccination coverage against yellow fever (YF) is limited and no vaccines are still available for most arboviral diseases, including dengue [1,12]. Therefore, the surveillance of *Aedes* vectors is crucial for preparedness, response, prevention and control of arboviral epidemics.

Côte d’Ivoire is one of the most important emerging and re-emerging foci of DEN and YF in Africa [13,14]. Four DENV serotypes (DENV1–4) and YFV are present there. In the recent years (2017–2024), the country has faced multiple DEN and YF outbreaks, with the majority (80–90%) of cases reported in the greater Abidjan metropolitan area (7 million inhabitants) [15–17]. For instance, DEN outbreaks caused 321 laboratory-confirmed cases and 27 deaths in 2023 [18,19], and 4,050 confirmed and 2 fatal DEN cases in 2024, showing that disease burden raised by 14 times [20]. These numbers likely represent only a fraction of the actual cases, as many may remain undiagnosed, unreported or misdiagnosed as malaria due to limited diagnostic capacity or surveillance sensitivity [2,21]. Nonetheless, over 80–90% of DEN and YF cases reported in Abidjan are recorded in the health district of Cocody-Bingerville (~900,000 inhabitants). DEN and YF outbreaks in Cocody-Bingerville mostly occur during rainy seasons [15–17]. Data highlight the ongoing and expanding threat of arboviral diseases in both urban and peri-urban areas [5].

In Cocody-Bingerville, urbanization is rapidly expanding into rural areas, thus resulting in many intra-urban and peri-urban villages. Despite this, as arboviral cases are predominantly reported in the urban hospitals, leading most studies on local *Aedes* vectors and arboviral risk assessment to focus primarily on the urban areas (cities with more advanced socio-economic infrastructures) and intra-urban villages (modern villages located inside of cities), often overlooking the peri-urban villages (rural villages situated on the outskirts of cities) [4]. Cocody-Bingerville has 22 villages (13 intra-urban and 9 peri-urban). However, local outbreak responses (e.g., removals of larval breeding sites, insecticide space spraying against *Aedes* larvae and adults, and local community awareness) are mostly implemented in the urban areas and intra-urban villages [13,14]. Though, numerous peri-urban villages are closely interconnected with and strongly influenced by these highly urbanized areas. Indeed, significant ground movement of people and materials (e.g., tires and cans) by vehicles occurs between the urban areas, the intra-urban villages and the peri-urban villages. These travels and goods exchange can facilitate the passive dispersal of attached eggs, larvae and adults of *Aedes* mosquitoes, and thereby contribute to arbovirus spread. However, the peri-urban villages often faced limited access to adequate water supply and waste management services, which are more readily available in urbanized areas. This lack of infrastructure can lead to the storage of water and the accumulation of unmanaged containers that can create breeding sites for the aquatic stages of *Aedes* mosquitoes [14,18]. Such conditions can increase the density of *Ae. aegypti* adult populations and biting rates, and the risk of DEN and YF epidemics in the peri-urban areas, similar to those observed in urban and intra-urban villages of Cocody-Bingerville [5]. Despite these risks, few vector control interventions (larviciding and adulticidicing programs) and no studies have examined *Aedes* vectors and arboviral epidemic risks in peri-urban villages compared with urban settings, including the intra-urban villages [4,5].

The present study aimed to assess and compare the patterns of *Ae. aegypti* larval infestation and container productivity and associated DEN and YF epidemic risks among peri-urban and intra-urban villages during the 2023–2024 DEN outbreaks in Cocody-Bingerville. We monitored *Aedes* immatures (eggs, larvae and pupae) and DEN and YF epidemic risks We used *Stegomyia* indices (house index: HI, container index: CI and Breteau index: BI) (qualitative approach) and pupal productivity indices (pupae per house index: PHI, pupae per container index: PCI and pupae per person index: PPI) (quantitative approach) [22,23]. We sampled *Aedes* mosquitoes in all study villages during the same months across seasonal variations (short rainy season, long rainy season, short dry season and long dry season). This study represents one of the first comparative studies of *Aedes* ecology and DEN and YF epidemic risks in peri-urban and intra-urban villages during a live DEN outbreak in Côte d’Ivoire.

## Methods

### Study area

**Fig 1:**
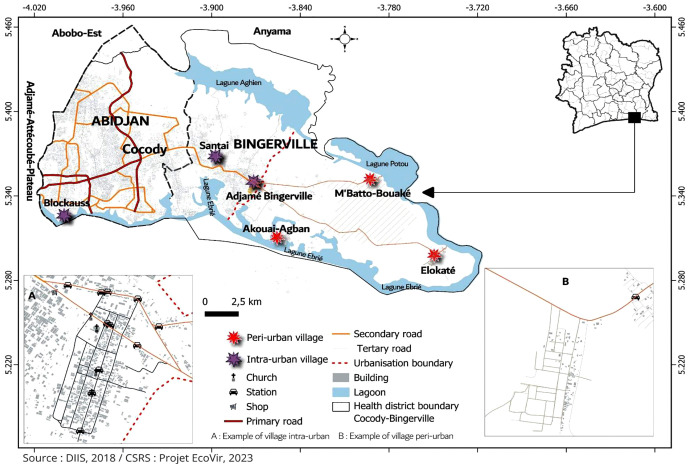
Location of the study sites in the health district of Cocody-Bingerville, southeastern Côte d’Ivoire from August 2023 to July 2024. The study sites were peri-urban villages and intra-urban villages of Cocody-Bingerville. The peri-urban villages comprise Akouai-Agban, M’batto-Bouaké and Elokaté characterized by secondary paved roads and mixture of green spaces, agricultural areas and spaced houses, and are located 5-15 km from the borders of the urban areas of Cocody-Bingerville. The intra-urban villages include Adjamé-Bingerville, Blockhauss and Santé that are all urban neighborhoods with numerous major and secondary paved roads and dense blocks of flats and located within the highly urbanized parts of the municipalities of Cocody and Bingerville. The study villages are within the boundaries of Cocody-Bingerville. Each study site include domestic and peridomestic ecozones. The map was created with QGIS software version 3.34 (https://www.qgis.org/),using the basemap is Openstreetmap data so, the basemap is open data, licensed under the Open Data Commons Open Database License (ODbL) by the OpenStreetMap Foundation (OSMF).

The study was carried out in peri-urban villages and intra-urban villages within the health district of Cocody-Bingerville (population ~ 897,000 inhabitants) in southeastern Côte d’Ivoire (Fig 1). This district is a part of the lagoon region administrated by the economic capital city of Abidjan (population ~ 7 million inhabitants), rapid and ongoing urban expansion characterizes the area, resulting in a heterogeneous landscape that includes numerous peri-urban and intra-urban settlements, from which the present study sites were selected. Cocody-Bingerville represents currently the major DEN and YF hotspot of the country, and harbor high densities of *Aedes* mosquitoes and abundant breeding sites [4,24,25]. Local arboviral incidences and *Ae. aegypti* population typically peak during the rainy seasons [4,24,25].

Cocody-Bingerville is in a coastal area with a tropical climate characterized by high temperatures and high RH throughout the year. The climate is marked by four rainfall-based seasons: long dry season (LDS) from December to March, long rainy season (LRS) from April to July, short dry season (SDS) from August to September, and short rainy season (SRS) from October and November. Average annual precipitation ranges from 1,200–2,400 mm. The annual temperature is approximately 26.5 °C. The annual RH ranges between 78 and 90%. Higher rainfall, temperature and RH are expected to increase density, larval development rate and survival of *Aedes* vectors.

### Study design

This study was conducted in three peri-urban villages and three intra-urban villages, totaling six study sites with roughly equal size. The study sites were selected among 22 villages (13 intra-urban and 9 peri-urban) located in Cocody-Bingerville. The peri-urban villages were represented by the villages of Akouai-Agban (5° 18’ 39” N, 3° 51’ 20” W), Elokaté (5° 17’ 38” N, 3° 44’ 56” W) and M’batto-Bouaké (5° 19’ 0” N, 3° 48’ 2” W) (Fig 1). All sites were located within a 15 km radius from the border of the urban health district of Cocody-Bingerville. The selected sites were characterized by a mixture of residential, commercial and agricultural land-use, with infrastructure limited to unpaved or secondary paved roads. The peri-urban villages (6,300 inhabitants) are less densely populated than the intra-urban areas (9,200 inhabitants) [26]. The intra-urban villages included Adjamé-Bingerville (5° 20’ 45” N, 3° 52’ 20” W), Blockhauss 5° 19’ 24” N, 4° 0’ 7” W) and Santé (5° 21’ 56” N, 3° 53’ 55” W) (Fig 1). These intra-urban villages were located within the urban municipalities of Cocody and Bingerville, and were characterized by advanced infrastructure, including residential and commercial buildings, primary and paved roads and presence of trade, industrial and administrative services. Each study site was subdivided into two ecological zones: domestic and peridomestic ecozones. Domestic ecozones were defined as spaces characterized by the presence of human-inhabited houses and associated structures. Peridomestic ecozones referred to areas extending up to 50 m surrounding the domestic ecozones. They included public or private spaces: roadsides, garages, schools, green spaces, agricultural fields and bushes.

Households were randomly selected systematically based on the willingness of the residents to participate, ensuring representative coverage of the entire village. When it was not possible to include a household, this household was replaced with a closest one. Collections were then conducted quarterly from August 2023 to July 2024, in the same months to minimize temporal biases. Cross-sectional surveys were conducted during SDS, LRS, SRS and LDS at each study site, resulting in a total of 24 collection events (12 in peri-urban and 12 in intra-urban villages).

### Egg collections

*Aedes* mosquito eggs were collected using standard WHO ovitrap method [27,28]. A total of 100 ovitraps (50 in domestic ecozone and 50 in peridomestic ecozone) was placed at each study site during each survey based on previous literature [23]. Therefore, 2,400 ovitraps (1,200 in peri-urban villages and 1,200 in intra-urban villages) were deployed. The ovitraps were cut-out metal and back-painted boxes with a volume of 400 cm^3^. Each ovitrap was garnished with hardboard paddles, rough on one side which served as an egg-laying substrate. The ovitraps were filled out at ¾ volume with a mixture of distilled water and rainwater (ratio: 1:1) to improve attractiveness for domestic and peridomestic egg-laying gravid *Aedes* females [27]. Ovitraps were left in the field for a one-week period during each survey [27]. They were suspended at 1.5 m above ground and covered to secure and protect with wire mesh against human or animal disturbance, sunlight and rainfall. All ovitrap contents (paddles, mosquito larvae or pupae and water) were collected and transferred separately into different plastic cups labelled (household code, ecozone type, study site and collection date).

### Larval and pupal collections

We searched for and sampled *Aedes* mosquito larvae and pupae among water-holding containers using dipping technique with a ladle or a pipette [25,29]. Collections were done in 100 households (i.e., in domestic and peridomestic ecozones) randomly selected per study site and per survey. All pupae were collected. The samples were stored separately in plastic cups labelled (household code, ecozone type, study site and collection date). All inspected breeding sites were characterized and classified into seven categories according to the WHO guidelines [30], as follows: 1) Large containers: drums and barrels (i.e., water volume > 50 liters); 2) Medium containers (i.e., buckets, larges pots, and small barrels: 10 liters ≤ volume < 50 liters); 3) Small containers (i.e., all container types: Water volume < 10 liters); 4) Tires (i.e., discarded bicycle, motorcycle, car, or other motor vehicle tires); 5) Water troughs (i.e., any type of container of any material used to feed animals); 6) Flowerpots (i.e., flower containers); and 7) Others (i.e., brick holes, shoes, tarpaulins, wooden boxes, mortar, sheet metal, leaf armpits, snail shells, puddles and tree holes). All the inspections were done by three trained teams. Each team was composed of two and the same persons: one sampled mosquito larvae or pupae and the other collected container data. Inspection lasted approximately for 10–15 min per household. The teams worked from 08:00–12:00 and 14:00–16:00. The teams rotated among the study sites to ensure similar sampling efforts and minimize biases.

### Laboratory procedures

All collected mosquito samples (eggs, larvae and pupae) were transported in cool boxes to our field laboratory in Bingerville. Each sample was put in plastic cup labelled with the sampling site, ecozone and date. The paddles were dried for seven days and then immersed separately in distilled water for egg hatching. All mosquito larvae were fed with Tetra-Min Baby Fish Food. Egg-drying and rearing of larvae and pupae to adults were performed under ambient conditions (temperature: 23.6–30.4ºC, RH: 67.1–82.5%, and light:dark photoperiod: 12:12 hours). The mortality during the rearing was low (eggs: 2.97%, larvae: 2.71% and pupae: 0.23%), thus making the comparison of emerged adults possible. All emerged adults were identified morphologically to species and sex under a binocular magnifying glass using available taxonomic keys of Cordelier et al. (OSTORM, N^O^33) [27], Rueda (Zootaxa 589) [31] and Huang (Zootaxa 700) [32]. Data were recorded in an entomological database designed.

### Climate data

Data on the climatic conditions, including rainfall, temperature and RH, were obtained from the Société d’Exploitation et de Développement Aéroportuaire, Aéronautique et Météorologique (SODEXAM) of Côte d’Ivoire (www.sodexam.com). We collected data monthly and averages used were disaggregated across the study region.

### Statistical analysis

Data were analyzed using R Studio version 4.4.2. A significance level of 5% was set. The container infestation rate (IR) was expressed as the percentage of containers with at least one *Ae. aegypti* larva or pupa (numerator) among the wet containers (denominator). The proportion of positive breeding site types (PP) was estimated as the percentage of each *Ae. aegypti*-positive container type (numerator) among the total *Ae. aegypti*-positive containers (denominator). Chi-square test (χ^2^) was used to compare IR and PP between the study areas.

*Aedes aegypti* oviposition indices such as ovitrap positivity index (OPI: percentage of *Ae. aegypti*-positive ovitraps among total number of ovitraps), mean egg count per ovitrap (MEO: mean number of *Ae. aegypti* eggs per number of ovitraps) and egg density index (EDI: mean number of *Ae. aegypti* eggs per *Ae. aegypti-*positive ovitraps) [33]. To deal with overdispersion due to excessive numbers of zeroes, the data were log-transformed [log (x + 1)], but the log-transformed data did not fulfil the normality and homoscedasticity conditions. Therefore, MEO and EDI indices were each compared between study areas and across seasons using generalized linear mixed model (GLMM) with negative binomial regression (Heteroscedasticity analysis using Breusch–Pagan test was significant, p < 0.0001). OPI indices were compared between study areas and over seasonality using logistic regression.

DEN and YF outbreak risks were determined using *Ae. aegypti* larval infestation levels based on *Stegomyia* indices that included container index (CI: percentage of *Ae. aegypti*-positive containers among water-holding containers), house index (HI: percentage of houses with at least one *Ae. aegypti*-positive container) and Breteau index (BI: number of *Ae. aegypti*-positive containers per 100 houses). HI, CI and BI were compared across the study areas using one-way analysis of variance (ANOVA), followed by Bonferroni adjustment. Moreover, HI, CI and BI values were compared with the World Health Organization (WHO)-established DEN and YF epidemic thresholds: DEN epidemic risk is high if CI > 3% or HI > 4% and BI > 5 [34]; and YF epidemic risk is low if CI < 3%, HI < 4% or BI < 5; moderate if 3% ≤ CI ≤ 20%, 4% ≤ HI ≤ 35% or 5 ≤ BI ≤ 50; and high if CI > 20%, HI > 35% or BI > 50 [35]. These *Stegomyia* indices were categorized as per the vector density index or density figure (DF) that ranges from 1 to 9 according to the WHO guidelines [35,36].

*Aedes aegypti* container productivity was estimated through pupal indices: pupae per house index (PHI: number of pupae divided by the number of houses), pupae per container index (PCI: number of pupae divided by the number of containers) and pupae per person index (PPI: number of pupae divided by the number of persons living in the houses). Additionally, as the *Ae. aegypti* pupal mortality was very low (<1%) and adult emergence was very high (>99%), human-biting index (HBI) was calculated as the number of females emerged from pupae divided by the number of persons dwelling in the inspected houses [37]. PHI, PCI, PPI and HBI were compared between the study areas using ANOVA, followed by Bonferroni post-hoc test.

We tested the relationships between *Ae. aegypti* indices (oviposition indices, *Stegomyia* indices, pupal indices and abundance) and climate variables (rainfall, temperature and RH) using the Sperman’s test [38]. We used the Sperman’s test because the data were not normally distributed (Shapiro-Wilk test was significant, p < 0.05). Sperman’s rank coefficient (ρ) measures the strength and the direction of association between two ranked variables. Values of ρ vary from −1–1. Correlation is very weak if |ρ| < 0.20; weak if 0.20 ≤ |ρ| ≤ 0.39; moderate if 0.40 ≤ |ρ| ≤ 0.59; strong if 0.60 ≤ |ρ| ≤ 0.79; and very strong if 0.80 ≤ |ρ| [38]. Correlation is negative if ρ < 0; null if ρ = 0; and positive if ρ > 0 [38]. Correlation is significant if p < 0.05, and non-significant if p > 0.05 [38].

### Ethics statement

Before sample collection, the study protocol received ethical approval from the national ethics committee of Côte d’Ivoire (N/Ref: 071–23/MSHPCMU/CNESVS-km). Permissions were obtained from the General Directorate of Health (GDH) of the Ministry of Health (MoH), the administrative and health authorities of Cocody-Bingerville and the local community leaders of the study areas. Sampling was done with the authorization of the residents and/or owners who provided oral or written consents. No financial or material incentives were given to participants. Personal data were anonymized and treated as confidential. This study did not involve endangered or protected species.

## Results

### Climate indicators

**Fig 2:**
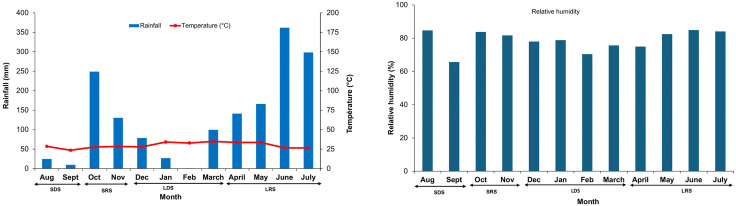
Monthly variations in climate indicators in the health district of Cocody-Bingerville, Côte d’Ivoire, from August 2023 to July 2024.

Rainfall, temperature and RH were high in the peri-urban and intra-urban villages (Fig 2 and S1 Table). The respective averages of rainfall, temperature and RH were 147.00 mm, 27.90 °C and 80.20%. These climatic variables varied significantly over seasons (rainfall: F = − 304.14, df = 3, p < 0.0001; temperature: F = 105.09, df = 3, p < 0.0001; and RH: F = 133.40, df = 3, p < 0.0001). Rainfall was higher during LRS (329.90 mm) and lower during SDS (17.05 mm). Temperature peaked during LDS (30.92 °C) and was lower during LRS (26.50 °C). RH was higher during LRS (84.50%) and lower during SDS (75.19%).

### Mosquito species composition

*Aedes aegypti* widely dominated *Aedes* genus in both peri-urban villages (98.05%, 13,157/13,419) and intra-urban villages (99.78%, 29,168/29,232) (Table 1). Taken together, *Ae. aegypti* proportion was 2.2-time lower in the peri-urban villages (31.09%, 13,157/42,325) compared to the intra-urban villages (68.91%, 29,168/42,325). Moreover, *Ae. aegypti* number was significantly lower in the peri-urban villages than in the intra-urban villages (χ^2^ = 81.570, df = 1, p < 0.0001). In the peri-urban villages, five additional *Aedes* species were found, among which *Aedes vittatus* (1.39%, 187/13,419) showed a proportion above 1%. Key non-*Aedes* mosquito taxa of public health importance, including the secondary vectors of arboviruses and predators or competitors with possible influential effects on local *Aedes* ecology were disproportionally sampled across the study areas. *Culex quinquefasciatus* and *Eretmapodites* species *(Eretmapodites chrysogaster* and *Eretmapodites quinquevittatus* were mostly collected in the peri-urban villages, while *Anopheles gambiae* malaria vector was mainly encountered in the intra-urban villages. Ecologically, species diversity was higher in the peri-urban villages (14 species) compared with the intra-urban villages (10 species). The greater species diversity in the peri-urban villages highlighted a key ecological insight as the collections yielded several predatory (*Eretmapodites*, *Lutzia* and *Toxorhynchites*) and sympatric (*Culex* and *Anopheles*) taxa (S2 Table).

**Table 1:** Species composition of *Aedes* mosquito adults emerged from eggs, larvae and pupae sampled in the peri-urban and intra-urban villages of Cocody-Bingerville, southeastern Côte d’Ivoire, from August 2023 to July 2024.

Village	Species	Egg				Larva				Pupae				Total			
		Female	Male	Total	%	Female	Male	Total	%	Female	Male	Total	%	Female	Male	Total	%
**Peri-urban**	*Aedes aegypti*	2768	2445	5213	99.58	2787	3061	5848	96.90	1032	1064	2096	97.53	6587	6570	13157	98.05
	*Aedes dendrophilus*	5	16	21	0.40	0	0	0	0	0	0	0	0	5	16	21	0.16
	*Aedes fraseri*	0	0	0	0	5	1	6	0.10	5	8	13	0.60	10	9	19	0.14
	*Aedes lilii*	0	0	0	0	8	12	20	0.33	0	0	0	0	8	12	20	0.15
	*Aedes luteocephalus*	0	0	0	0	2	13	15	0.25	0	0	0	0	2	13	15	0.11
	*Aedes vittatus*	1	0	1	0.02	70	76	146	2.42	24	16	40	1.86	95	92	187	1.39
	**Total**	**2774**	**2461**	**5235**	**100**	**2872**	**3163**	**6035**	**100**	**1061**	**1088**	**2149**	**100**	**6707**	**6712**	**13419**	**100**
**Intra-urban**	*Aedes aegypti*	4410	4862	9272	99.99	6935	6730	13665	99.80	2882	3349	6231	99.43	14227	14941	29168	99.78
	*Aedes fraseri*	0	0	0	0	21	6	27	0.20	1	2	3	0.05	22	8	30	0.10
	*Aedes vittatus*	0	1	1	0.01	0	0	0	0	8	25	33	0.53	8	26	34	0.12
	**Total**	**4410**	**4863**	**9273**	**100**	**6956**	**6736**	**13692**	**100**	**2891**	**3376**	**6267**	**100**	**14257**	**14975**	**29232**	**100**

%: percentage.

### *Aedes aegypti* population dynamics

#### Population abundance.

*Aedes aegypti* abundance varied across the ecozones and seasons in the peri-urban and intra-urban villages. Proportions were statistically higher in the domestic ecozones compared with the peridomestic ecozones in the peri-urban villages (61.94% *vs.* 38.06%; χ^2^ = 1498.8, df = 1, p < 0.0001), but comparable between both ecozone types in the intra-urban villages (49.95% *vs.* 50.05%; χ^2^ = 0.0659, df = 1, p = 0.7974). Proportions in the domestic ecozones were higher in the peri-urban villages than that in the intra-urban villages (61.94% *vs.* 49.95%; χ^2^ = 523.87, df = 1, p < 0.0001). Conversely, *Ae. aegypti* proportions in the peridomestic ecozones were higher in the intra-urban villages compared with the peri-urban villages (50.05% *vs.* 38.06%; χ^2^ = 523.87, df = 1, p < 0.0001) (S3 Table).

**Fig 3:**
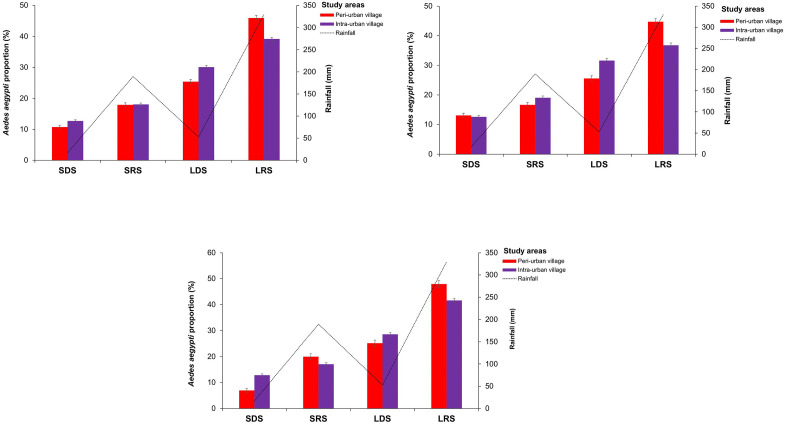
Seasonal variations in the proportions of *Aedes aegypti* adults emerged from eggs, larvae and pupae sampled in the peri-urban and intra-urban villages of Cocody-Bingerville, southeastern Côte d’Ivoire, from August 2023 to July 2024. A: Overall, B: Domestic ecozone, C: Peridomestic ecozone. LDS: long dry season, LRS: long rainy season, SDS: short dry season, SRS: short rainy season. Error bars show confidence intervals (95% CI).

**Fig 4:**
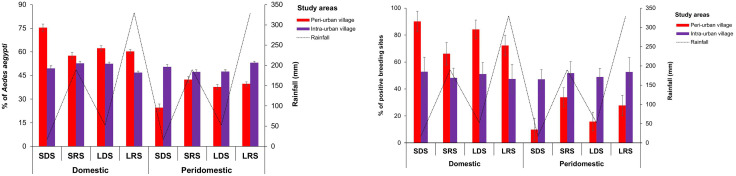
Geographical variations in *Aedes aegypti* proportions and breeding site infestation in the domestic and peridomestic ecozones in the peri-urban and intra-urban villages of Cocody-Bingerville, southeastern Côte d’Ivoire, from August 2023 to July 2024. A: *Aedes aegypti* proportion, B: Breeding site proportion. LDS: long dry season, LRS: long rainy season, SDS: short dry season, SRS: short rainy season. Error bars show confidence intervals (95% CI).

*Ae. aegypti* proportions peaked during LRS in both peri-urban villages (45.95%) and intra-urban villages (39.19%) (χ^2^ = 170.56, df = 1, p < 0.0001), as well as in the domestic and peri-domestic ecozones of all villages (Fig 3). The lowest proportions were observed during SDS in the peri-urban villages (10.75%) and the intra-urban villages (12.70%) (χ^2^ = 32.308, df = 1, p < 0.0001) (Fig 3). Across seasons, proportions were significantly higher in the domestic ecozones compared with the peridomestic ecozones in the peri-urban villages (all p < 0.05), but statistical similar between the two ecozones in the intra-urban villages (all p > 0.05) (Fig 4).

#### Larval infestation.

IR was significantly lower in the peri-urban villages than in the intra-urban villages (29.96% *vs.* 36.71%; χ^2^ = 17.534, df = 1, p < 0.0001) (S4 Table). All the seven types of containers were found infested with *Ae. aegypti* immatures in both peri-urban and intra-urban villages (S1A Fig). Tires, flowerpots, small containers and medium containers were the most infested items in all villages. Overall, all container types were *Ae. aegypti*-positive in both domestic and peridomestic ecozones in both village types (S1B and S1C Figs), except for water troughs in the peridomestic ecozones (S1C Fig). IR peaked during LRS in both peri-urban villages (33.86%) and intra-urban villages (42.56%). However, the lowest IR values were still high during dry seasons in both peri-urban villages (SDS: 25.82%) and intra-urban villages (LDS: 30.62%).

**Fig 5:**
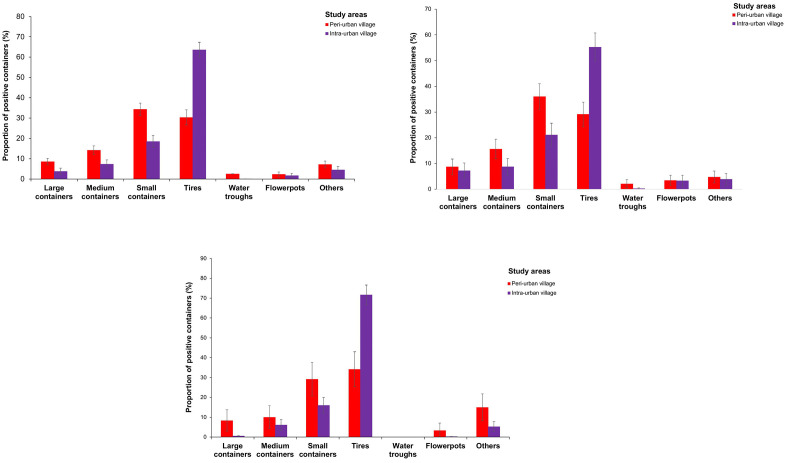
Proportion of *Aedes aegypti* breeding sites in the peri-urban and intra-urban villages of Cocody-Bingerville, southeastern Côte d’Ivoire, from August 2023 to July 2024. A: Overall, B: Domestic ecozone, C: Peridomestic ecozone. Error bars indicate confidence intervals (95% CI). Others includes breeding containers made with brick holes, shoes, tarpaulins, wooden boxes, mortar, sheet metal, leaf armpits snail shells, underground puddles and tree holes.

The distribution of *Ae. aegypti*-positive container types and proportions differed significantly between peri-urban and intra-urban areas, as well as domestic and peridomestic ecozones (χ^2^ = 267.5, df = 3, p < 0.0001). Pooled together, PP was significantly lower in the peri-urban villages (PP = 42.44%, 497/1,171) than in the intra-urban villages (PP = 57.56%, 675/1,171) (χ^2^ = 52.905, df = 1, p < 0.0001). Small containers (PP = 34.41%), tires (PP = 30.38%) and medium containers (PP = 14.29%) were mostly abundant in the peri-urban villages, while tires (PP = 63.65%) and small containers (PP = 18.55%) dominated in the intra-urban villages (Fig 5A). PP was 3-time and significantly higher in the domestic ecozones (75.55%, 377/497) compared to the peridomestic ecozones (24.14%) in the peri-urban villages (χ^2^ = 263.73, df = 1, p < 0.0001), but nearly equal between the two ecozones (49.11% *vs.* 50.89%, respectively) in the intra-urban villages (χ^2^ = 0.3591, df = 1, p = 0.549). Large, medium, and small containers, as well as water troughs, were more frequently observed in the domestic ecozones (Fig 5B), while tires and other container types were more common in the peridomestic ecozones (Fig 5C). Seasonal patterns showed more pronounced variability in the peri-urban villages, with positive containers’ proportions peaking during SDS in the domestic ecozones and during SRS in the peridomestic ecozones. In contrast, the peri-urban villages showed relatively similar proportions of positive containers in both domestic and peridomestic ecozones across seasons. Compared with the intra-urban villages, the peri-urban villages yielded consistently more positive containers in the domestic ecozones, but a lower number in the peridomestic ecozones across all seasons (S5 Table).

#### Container productivity.

Containers produced 3-fold and significantly lower number of *Ae. aegypti* pupae in the peri-urban villages (2,096 pupae) than in the intra-urban villages (6,231 pupae) (χ^2^ = 4104.7, df = 1, p < 0.0001) (Table 2). In the peri-urban villages, the most productive containers were small containers (31.11%), followed by tires (30.53%) and medium containers (20.13%), providing together 81.77% of the total pupae (S2A Fig). In the intra-urban villages, the key containers were tires (64.45%) and small containers (18.75%) that yielded 83.20% of the total pupae (S2A Fig).

**Table 2:** Seasonal variations in container productivity for *Aedes aegypti* pupae among peri-urban and intra-urban villages of Cocody-Bingerville, southeastern Côte d’Ivoire from August 2023 to July 2024.

Village	Container	Short dry season		Short rainy season		Long dry season		Long rainy season		Total	
		n	%	n	%	n	%	n	%	n	%
**Peri-urban**	Large containers	3	1,25	53	11,47	48	8,5	25	3,02	129	6,15
	Medium containers	25	10,42	68	14,72	65	11,5	264	31,85	422	20,13
	Small containers	74	30,83	129	27,92	225	39,82	224	27,02	652	31,11
	Tires	123	51,25	176	38,1	145	25,66	196	23,64	640	30,53
	Water troughs	15	6,25	6	1,3	0	na	21	2,53	42	2
	Flowerpots	0	na	0	na	33	5,84	16	1,93	49	2,34
	Others	0	na	30	6,49	49	8,67	83	10,01	162	7,73
	**Total**	**240**	**100**	**462**	**100**	**565**	**100**	**829**	**100**	**2096**	**100**
**Intra-urban**	Large containers	74	12,13	36	3,09	21	1,07	142	5,7	273	4,38
	Medium containers	36	5,9	59	5,07	128	6,52	162	6,5	385	6,18
	Small containers	65	10,66	125	10,74	439	22,35	535	21,46	1164	18,68
	Tires	421	69,02	860	73,88	1310	66,7	1433	57,48	4024	64,58
	Water troughs	8	1,31	0	na	23	1,17	0	na	31	0,5
	Flowerpots	0	na	0	na	5	0,25	106	4,25	111	1,78
	Others	6	0,98	84	7,22	38	1,93	115	4,61	243	3,9
	**Total**	**610**	**100**	**1164**	**100**	**1964**	**100**	**2493**	**100**	**6231**	**100**

*%: percentage of pupae, n: number of pupae, na: not applicable. Others includes breeding containers made up of brick holes, Shoes, tarpaulins, wooden boxes, mortar, sheet metal, leaf armpits, snail shells, underground puddles and tree holes.*

#### Ecozonal shift in container productivity.

Container productivity differed significantly across the ecozones in all the study villages (χ^2^ = 742.89, df = 3, p < 0.0001). In the peri-urban villages, the most productive containers (productivity > 10% of pupae) were mainly found in the domestic ecozones (70.94%, 1,487/2,096) compared with the peridomestic ecozones (29.06%, 609/2,096) (χ^2^ = 733.9, df = 1, p < 0.0001) (S5 Table). They were small containers (36.72% *vs*. 17.41%), tires (31.14% *vs.* 29.06%) and medium containers (15.94% *vs.* 30,38%) in the domestic and peridomestic ecozones (S2B and S2C Figs). In the intra-urban villages, container productivity was nearly similar between the domestic ecozones (48.8%, 3,040/6,231) and peridomestic ecozones (51.2%, 3,191/6,231) (χ^2^ = 7.222, df = 1, p < 0.0072), with tires and small containers as the most productive habitats in both ecozones (S6 Table).

#### Seasonal shift in container productivity.

Container productivity was significantly influenced by seasonal variability in all the study villages (χ^2^ = 2247.5, df = 7, p < 0.0001). The highest proportions of pupae were recorded in LRS in all the study villages, representing 39.55% (829/2,096) in the peri-urban villages and 40.01% (2,493/6,231) in the intra-urban villages (Table 2). During this period, container productivity was 3-fold lower in the peri-urban villages (829 pupae) than the intra-urban villages (2,493 pupae) (Table 2). During LRS, the most productive containers in the peri-urban villages were medium containers (31.85%), small containers (27.02%) and tires (23.64%), all cumulating 82.51% of pupae. During this season, tires alone provided more than half of pupal production (57.48%) in addition to small containers (21.46%), both producing 78.94% of the pupae in the intra-urban villages. The lowest proportions of pupae were found during SDS in the peri-urban villages (11.45%, 240/2,096) and the intra-urban villages (9.79%, 610/6,231). During SDS, the highest pupal productivity was found in tires (51.25%), followed by small containers (30.83%) and medium containers (10.42%) in the peri-urban villages. Similarly, during the same season, tires produced the highest proportion of pupae (69.02%), followed by large containers (12.13%) and small containers (10.66%) in the intra-urban villages (Table 2). During SDS, these key containers yielded 92.50% and 91.80% of pupae in the peri-urban and intra-urban villages, respectively (Table 2). Container productivity during LDS was significantly higher compared with that found during SDS in the peri-urban villages (χ^2^ = 160.56, df = 1, p < 0.0001) and the intra-urban villages (χ^2^ = 896.33, df = 1, p < 0.0001), and that recorded during SRS in both peri-urban (χ^2^ = 13.165, df = 1, p < 0.0001) and intra-urban (χ^2^ = 272.49, df = 1, p < 0.0001) villages (Table 2). Similar seasonal patterns were observed in the domestic and peridomestic ecozones in the peri-urban and intra-urban villages, but with pronounced magnitude in the domestic ecozones.

### *Aedes aegypti* indices

#### Oviposition indices.

OPI, MEO and EDI values were 39.95% [37.08%, 42.88%], 4.64 [4.08, 5.19] egg/ovitrap/week and 11.61 [10.49, 12.70] egg/ovitrap/week in the peri-urban villages, and (53.04% [50.04%, 56.04%]), 8.67 [7.89, 9.46] egg/ovitrap/week and 16.35 [15.19, 17.51] egg/ovitrap/week) in the intra-urban villages, respectively (S7 Table). GLMM showed that all the *Ae. aegypti* oviposition indices were statistically lower in the peri-urban villages than in the intra-urban villages (OPI: Estimate = −0.5295, Z = −6.13, p < 0.0001, OR = 0.59; MEO: Estimate = −0.6260, Z = −6.332, p < 0.0001; and EDI: Estimate = −0.3425, Z = −5.99, p < 0.0001). All oviposition indices were higher in the domestic ecozones than in the peridomestic ecozones in the peri-urban villages (all p < 0.05), but similar between the two ecozones in the intra-urban villages (all p > 0.05) (S8 Table). The three indices inconsistently varied over the seasons. OPI, MEO and EDI peaked in LDS, LDS and SRS in the peri-urban villages, and LDS, LRS and LRS in the intra-urban villages, respectively. OPI, MEO and EDI displayed the lowest values, respectively, during SRS, SDS and SDS in the peri-urban villages, and SRS, SRS and LDS in the intra-urban villages (S7 Table).

#### Stegomyia indices and epidemic risks of dengue and yellow fever.

**Fig 6:**
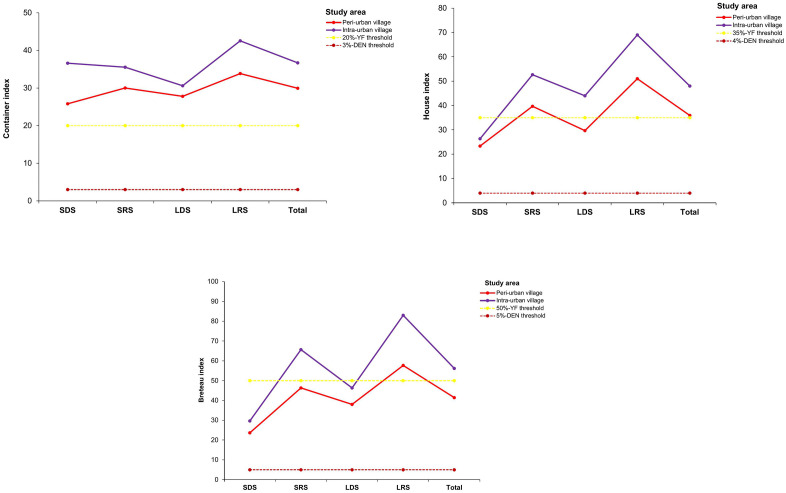
Seasonal variations of *Stegomyia* indices and epidemic risks of dengue and yellow fever in the peri-urban and intra-urban villages of Cocody-Bingerville, southeastern Côte d’Ivoire, from August 2023 to July 2024. %: percentage. Error bars show confidence intervals (95% CI). A: Container index, B: House index, C: Breteau index. LDS: long dry season, LRS: long rainy season, SDS: short dry season, SRS: short rainy season. Dengue epidemic thresholds are 4% for house index, 3% for container index and 5 for Breteau index [34]. Yellow fever epidemic thresholds are 35% for house index, 20% for container index and 50 for Breteau index [35].

Values of *Stegomyia* indices exceeded WHO thresholds in both peri-urban and intra-urban villages and showed low statistical differences between village types (p > 0.05) (Fig 6). CI values were 29.96% [27.88%, 32.39%] in the peri-urban villages and 36.71% [34.50%, 38.96%] in the intra-urban villages (Fig 6A). HI values were estimated at 35.92% [32.63%, 38.11%] and 48.00% [45.14%, 50.87%] in the peri-urban and intra-urban villages, respectively (Fig 6B). BI values were 41.42 [38.04, 43.68] in the peri-urban villages and 56.17 [53.31, 59.00] in the intra-urban villages (Fig 6C). All *Stegomyia* indices were statistically comparable among the peri-urban and intra-urban villages (CI: F = 2.95, df = 1, p = 0.0999; HI: F = 3.072, df = 1, p = 0.0936; and BI: F = 2.602, df = 1, p = 0.121). No significant variation was observed for the CI index (F = 0.856; df = 7; p = 0.56), whereas the HI and BI indices varied significantly across the seasons in all the focus villages (HI: F = 5.227, df = 1, p = 0.0029; BI: F = 4.028, df = 1, p = 0.0099). CI displayed the highest values during LRS in both peri-urban (33.86% [29.76%, 38.14%]) and intra-urban (42.56% [38.52%, 46.69%]) villages. The lowest values of CI were observed during SDS (25.82% [20.75%, 31.42%]) in the peri-urban villages and during LDS (30.62% [24.41%, 35.08%]) in the intra-urban villages. HI showed peaks during LRS (51.00% [45.19%, 56.79%]) in the peri-urban villages and LRS (69.00% [63.43%, 74.19%]) in the intra-urban villages, that were statistically comparable (F = 18, df = 1, p = 0.5580). HI decreased gradually and exhibited lower values during SDS in the peri-urban (23.33% [18.66%, 28.54%]) and intra-urban (26.33% [21.44%, 31.70%]) villages. BI values peaked during LRS in the peri-urban villages (57.67 [51.67, 63.32]) and the intra-urban villages (83.00 [78.28, 87.07]). BI displayed the lower values during SDS in both peri-urban villages (23.67 [18.97, 28.89]) and intra-urban villages (29.67 [24.55, 35.19]).

Epidemic risk levels associated with *Stegomyia* indices were high for DEN and varied from moderate to high for YF in the peri-urban and intra-urban villages (Table 3). Overall, all CI, HI and BI values in the peri-urban and intra-urban villages exceeded the WHO DEN and YF epidemic thresholds [34,35], except for BI for YF in the peri-urban villages. The risk indices corresponded to DFs of 5–8 for the peri-urban villages and 6–8 for the intra-urban villages and revealed high DEN and YF epidemic risk levels in all the study villages. Although DEN epidemic risk levels exhibited seasonal variations in all the peri-urban and intra-urban villages, values were permanently high and above the WHO DEN epidemic thresholds in all the study villages during the whole study period. YF epidemic risks were generally high in all the study villages during almost all seasons, but moderate during LDS and SDS in the peri-urban villages and SDS in the intra-urban villages.

**Table 3:** *Stegomyia* indices and levels and dengue and yellow fever epidemic risks in the peri-urban and intra-urban villages of Cocody-Bingerville, southeastern Côte d’Ivoire, from August 2023 to July 2024.

Village	Season	Container		House		Container index (%)		House index (%)		Breteau index		Density figure	Risk level	
		Wet	Positive	Inspected	Positive	Mean	95% CI	Mean	95% CI	Mean	95% CI		DEN	YF
**Peri-urban**	SDS	275	71	300	70	25.82	[20.75-31.42]	23.33	[18.66-28.54]	23.67	[18.97-28.89]	4-7	High	Moderate
	SRS	463	139	300	119	30.02	[26.24-35.14]	39.67	[31.84-43.08]	46.33	[38.30-49.82]	5-8	High	High
	LDS	410	114	300	89	27.80	[23.52-32.41]	29.67	[24.55-35.19]	38.00	[32.48-43.76]	5-7	High	Moderate
	LRS	511	173	300	153	33.86	[29.76-38.14]	51.00	[45.19-56.79]	57.67	[51.67-63.32]	6-8	High	High
	**Total**	**1659**	**497**	**1200**	**431**	**29.96**	**[27.88-32.39]**	**35.92**	**[32.63-38.11]**	**41.42**	**[38.04-43.68]**	**5-8**	**High**	**High**
**Intra-urban**	SDS	243	89	300	79	36.63	[30.55-38.96]	26.33	[21.44-31.70]	29.67	[24.55-35.19]	4-8	High	Moderate
	SRS	554	197	300	158	35.56	[31.57-39.70]	52.67	[46.85-58.43]	65.67	[59.99-71.03]	6-8	High	High
	LDS	454	139	300	132	30.62	[24.41-35.08]	44.00	[38.30-49.82]	46.33	[40.58-52.16]	5-8	High	High
	LRS	585	249	300	207	42.56	[38.52-46.69]	69.00	[63.43-74.19]	83.00	[78.26-87.07]	5-9	High	High
	**Total**	**1836**	**674**	**1200**	**576**	**36.71**	**[34.50-38.96]**	**48.00**	**[45.14-50.87]**	**56.17**	**[53.31-59.00]**	**6-8**	**High**	**High**

%: percentage, DEN: dengue, YF: yellow fever, CI: confidence interval. LDS: long dry season, LRS: long rainy season, SDS: short dry season, SRS: short rainy season. *Stegomyia* indices are based on container infestation with *Aedes aegypti* larvae or pupae. WHO: World Health Organization. Density figure and DEN and YF epidemic risk level are estimated according to the WHO guidelines [34,36].

#### Pupal indices.

PCI and PHI respective values were significantly lower in the peri-urban villages (1.26 [1.02, 1.51] pupae/container and 1.75 [1.45, 2.05] pupae/house) compared with the intra-urban villages (3.39 [3.32, 3.46] pupae/container and 5.19 [4.44, 5.93] pupae/house) (PCI: F = 5.8369, df = 1, p < 0.0001; and PHI: F = 5.894, df = 1, p < 0.0001) (Table 4). However, PPI and HBI were, respectively, 2.3 and 2.1-time higher in the intra-urban villages (1.25 [1.11, 1.30] pupae/person and 0.27 [0.25, 0.28] female/person) than in the peri-urban villages (0.54 [0.52, 0.56] pupae/person and 0.58 [0.56, 0.59] female/person), suggesting that intra-urban offered more suitable conditions for *Ae. aegypti* development and was more exposed to arbovirus transmission risk.

**Table 4:** *Aedes aegypti* pupal indices in the peri-urban and intra-urban villages of Cocody-Bingerville, southeastern Côte d’Ivoire, from August 2023 to July 2024.

Village	Season	Wet container	House	Person	Pupae	Female	PCI		PHI		PPI		HBI	
							Mean	95% CI	Mean	95% CI	Mean	95% CI	Mean	95% CI
**Peri-urban**	SDS	275	300	1053	240	143	0.87	[0.83-0.91]	0.80	[0.17-1.43]	0.23	[0.20-0.25]	0.14	[0.12-0.16]
	SRS	463	300	715	462	203	0.99	[0.98-1.00]	1.54	[0.73-2.35]	0.65	[0.61-0.68]	0.28	[0.25-0.32]
	LDS	410	300	1076	565	288	1.38	[1.32-1.44]	1.88	[0.67-3.10]	0.53	[0.49-0.56]	0.27	[0.24-0.30]
	LRS	511	300	1032	829	398	1.62	[1.55-1.69]	2.76	[1.31-4.22]	0.80	[0.78-0.83]	0.39	[0.36-0.42]
	**Total**	**1659**	**1200**	**3876**	**2096**	**1032**	**1.26**	**[1.01-1.51]**	**1.75**	**[1.45-2.05]**	**0.54**	**[0.52-0.56]**	**0.27**	**[0.25-0.28]**
**Intra-urban**	SDS	243	300	1316	610	281	2.51	[2.36-2.66]	2.03	[1.17-2.90]	0.46	[0.44-0.49]	0.21	[0.19-0.24]
	SRS	554	300	1191	1164	527	2.10	[2.01-2.19]	3.88	[2.64-5.12]	0.98	[0.97-1.00]	0.44	[0.41-0.47]
	LDS	454	300	1211	1964	920	4.33	[4.13-4.47]	6.55	[4.85-8.18]	1.62	[1.57-1.65]	0.76	[0.73-0.78]
	LRS	585	300	1276	2493	1154	4.26	[4.11-4.41]	8.31	[6.45-10.2]	1.95	[1.90-2.00]	0.90	[0.89-0.92]
	**Total**	**1836**	**1200**	**4994**	**6231**	**2882**	**3.39**	**[3.32-3.46]**	**5.19**	**[4.44-5.93]**	**1.25**	**[1.11-1.30]**	**0.58**	**[0.56-0.59]**

PCI: pupae per container index, PHI: pupae per house index, PPI: pupae per person index, HBI: human biting index, CI: confidence interval. LDS: long dry season, LRS: long rainy season, SDS: short dry season, SRS: short rainy season. Female is *Aedes aegypti* females. HBI is estimated as the mean number of *Aedes aegypti* females per person.

All the pupal indices followed the seasonal variations in all the study villages. PCI peaked during LRS in the peri-urban villages (1.62 [1.55, 1.69] pupae/container) and LDS in the intra-urban villages (4.33 [4.13, 4.47] pupae/container). PCI reached the lowest values during SDS (0.87 [0.83, 0.91] pupae/container) in the peri-urban and SRS (2.10 [2.01, 2.19] pupae/container) in the intra-urban villages. PHI exhibited peaks during LRS in both peri-urban (2.76 [1.31, 4.22] pupae/house) and intra-urban (8.31 [6.45, 10.20] pupae/house) villages, and reached the lowest values during SDS in the peri-urban villages (0.80 [0.17, 1.43] pupae/house) and intra-urban villages (2.03 [1.17, 2.90] pupae/house). PPI peaks occurred simultaneously during LRS in both peri-urban villages (0.80 [0.78, 0.83] pupae/person) and intra-urban villages (1.95 [1.90, 2.00] pupae/person). PPI decreased considerably across seasonal variations and reached the lowest values during SDS in the peri-urban villages (0.23 [0.20, 0.25] pupae/person) and the intra-urban villages (0.46 [0.44, 0.49] pupae/person). HBI peaked during LRS in the peri-urban villages (0.39 [0.36, 0.42] female/person) and the intra-urban villages (0.90 [0.89, 0.92] female/person). HBI decreased over the seasons and showed the lowest values during SDS in both peri-urban villages (0.14 [0.12, 0.16] female/person) and in intra-urban villages (0.21 [0.19, 0.24] female/person)*.* Although the intra-urban villages had higher *Ae. aegypti* vector densities, the peri-urban villages still sustained significant risk, showing that both contributed to DENV transmission.

### Climate effects on *Aedes aegypti* abundance and arbovirus transmission risks

Overall, climate effects and correlations with *Ae. aegypti* abundances were stronger in the peri-urban villages than in intra-urban villages. We found weak, but positive and statistically significant correlations between *Ae. aegypti* numbers and rainfall in the peri-urban villages (ρ = 0.1913, p < 0.0001) and the intra-urban villages (ρ = 0.068, p < 0.0001). *Ae. aegypti* numbers and temperature exhibited negative and weak associations that were significant in the peri-urban villages (ρ = − 0.1474, p < 0.0001), but not significant in the intra-urban villages (ρ = − 0.0219, p = 0.4394). *Ae. aegypti* numbers and RH showed weak and non-significant correlations which were positive in the peri-urban villages (ρ = 0.008, p = 0.8052) and negative in the intra-urban villages (ρ = −0.0053, p = 0.8528). Rainfall was more correlated with *Ae. aegypti* number than temperature or RH, suggesting that it is a useful early warning index for vector surges in both peri-urban and intra-urban settings.

Among *Stegomyia* indices, HI and BI were positively, strongly and significantly correlated with rainfall in the peri-urban and intra-urban villages (all p < 0.05) (S9 Table). For pupal indices, PHI (ρ = 0.5948, p = 0.0414), PPI (ρ = 0.6478, p = 0.0228) and HBI (ρ = 0.5959, p = 0.0409) were each positively, moderately and significantly correlated with rainfall in the peri-urban villages. However, the correlations between rainfall and all pupal indices were positive, weak and non-significant in the intra-urban villages (all p > 0.05) (S9 Table). Temperature was negatively, weakly and non-significantly associated with all *Stegomyia* indices and all pupal indices in all the study villages (all p > 0.05), except for HI in the peri-urban villages and HI and BI in the intra-urban villages (S6 Table). Overall, there were negative, weak and non-significant relationships between RH and all *Stegomyia* indices and all pupal indices in the peri-urban and intra-urban villages (all p > 0.05) (S9 Table).

## Discussion

### Summary of main findings

This study provides a first comparative analysis of *Ae. aegypti* populations and epidemic risks of DEN and YF in the peri-urban and intra-urban villages during disease outbreaks, within the main hotspots in Cocody-Bingerville [12,13,17,20]. While previous entomological investigations and outbreak responses have predominantly focused on urban neighborhoods, including intra-urban villages [4,5], peri-urban villages have remained understudied. The present study showed that the peri-urban and intra-urban villages harbor high numbers of *Ae. aegypti* immatures and larval breeding containers. As a result, both peri-urban and intra-urban villages showed high *Stegomyia* indices that exceeded the WHO DEN and YF epidemic thresholds. Therefore, peri-urban villages should be integrated into vector control plans and arboviral outbreak responses. However, peri-urban and intra-urban villages exhibited entomo-epidemiological similarities and dissimilarities that are discussed below.

### Ecology of *Aedes* vectors

Holistically, our data showed that *Ae. aegypti* was the most abundant *Aedes* species in both peri-urban villages (>98%) and intra-urban villages (~100%). *Ae. aegypti* dominance in these villages could be explained by high numbers of people and artificial containers (i.e., tires, small containers and medium containers that produced over 80% of pupae), as reported in the cities of Abidjan and Ouagadougou, Burkina Faso [5,39]. *Ae. aegypti* is highly anthropophagic and preferentially feeds on humans and breeds in man-made containers [5,39]. Moreover, tropical climatic conditions (i.e., high precipitations, high temperatures and high RH) recorded in the present study areas are highly suitable for *Ae. aegypti* oviposition and larval development to adults [8,40].

At reductionist point, this study revealed several entomological and ecological differences between the peri-urban and intra-urban villages. Overall, mosquito species richness was higher in the peri-urban villages (14 species) compared with the intra-urban villages (10 species). This species compositional difference could be explained by a mixture of urbanized, agricultural and natural land-covers (buildings, farms and bushes) in the peri-urban villages and the dominance of urban environments in intra-urban villages. The peri-urban villages harbor aquatic habitats that host larvae of sympatric species (e.g., *Cx. quinquefasciatus* and *An. gambiae*) and predatory taxa (e.g., *Eretmapodites*, *Lutzia* and *Toxorhynchites*) that could raise the biodiversity of local *Aedes* species. The peri-urban villages harbor five additional wild *Aedes* species (*Ae. dendrophilus*, *Ae. fraseri, Ae. lilii*, *Ae. luteocephalus* and *Ae. vittatus*) that could increase local arbovirus transmission risks. The regression of predatory and competitive species in the intra-urban villages increases *Ae. aegypti* density that was 2-fold higher compared with the intra-urban villages. However, this *Ae. aegypti*-biased risk in the intra-urban villages could be compensated in the peri-urban villages by the presence of the wild and secondary *Aedes* vectors [35]. PPI and HBI were equal despite lower PHI/PCI in the peri-urban villages, suggesting probable compensatory mechanisms, such as higher survivorship or feeding rates that could increase local *Ae. aegypti* vectorial capacity.

### Ecozonal and seasonal variations of *Aedes aegypti* abundance

In this study*, Ae. aegypti* abundance differs considerably across ecozones in both peri-urban and intra-urban villages. Indeed, *Ae. aegypti* proportions were much higher in the domestic ecozones in the peri-urban villages, but similar between the domestic and peridomestic ecozones in the intra-urban villages. These disproportional distributions overlapped consistently with geographical presences of container productivity: 71% of pupae sampled in the domestic ecozones in the peri-urban villages, and 50% of pupae collected in each of the domestic and peridomestic ecozones in the peri-urban villages [4,5]. In all villages, containers found in the domestic ecozones were mostly water receptables and solid waste dispersed among households [4,5,41]. The peridomestic ecozones of the peri-urban villages had less productive containers (<29%) because these ecozones were mostly agricultural areas, dense vegetations and bushes. However, the peridomestic ecozones in the intra-urban villages had higher number of productive containers (e.g., used tires, cans, discarded items) abandoned or thrown in unmanaged premises (e.g., roadsides, garages, workplaces, schools, markets, green spaces and vegetation) due to poor environmental hygiene and sanitation services [23]. These peri-domiciliary vicinities are less exposed to anthropogenic disturbance, provide more stable habitats protected by vegetation and shade, and offer suitable microclimate and microbial food sources for the best development of *Aedes* larvae to pupae [4,5,23]. The priority areas for vector control efforts should be domestic ecozones in the peri-urban and both domestic and peri-urban ecozones in the intra-urban villages.

Our data also demonstrated that *Ae. aegypti* abundance shifts profoundly over seasonality in the peri-urban and intra-urban villages. Importantly, rainfall appears to be the main driver of *Ae. aegypti* population dynamics in all the villages, as reported in many African settings [29,42]. Rainfall was more correlated with *Ae. aegypti* densities than temperature and RH, suggesting that it is a useful early warning index for vector surges in the peri-urban and intra-urban areas. However, rainfall effects were stronger in the peri-urban villages compared with intra-urban villages. This could be possibly explained by higher receptacle exposure to rainwater in the peri-urban villages because access to potable water is limited there due to poor supply infrastructures and people collect rainwater to supplement their needs. Moreover, precipitations flood used and unused containers and provide multitude aquatic habitats for *Ae. aegypti* egg-laying and larval development to pupae, resulting in higher pupal productivity recorded during LRS in all villages [43,44]. In contrast, the lowest container productivity (<11%) was recorded during SDS in all the villages where only few container types (tires, small containers, and medium containers) produced over 90% of pupae sampled in this period. The drop in container productivity during SDS might be attributed to a dry out of containers associated with a decline of rainfall and the drought [45]. Unexpectedly, *Ae. aegypti* abundance and container productivity were significantly higher during LDS compared with SDS and SRS in all villages, and this could be explained by the complex effects of the interactions between climate and socio-behaviors on *Ae. aegypti* breeding patterns, as observed in Equator [46,47]. Indeed, during dry periods, people store water much more and for extended duration to compensate for low rainfall and water shortage [4,5]. This long-term water storage practice during LDS may support *Ae. aegypti* larval development in the containers. However, the decline of container productivity during SDS makes it the appropriate period for focused larval source management (LSM) targeting productive containers for maximum efficiency.

### Dengue and yellow fever epidemic risks

All the *Stegomyia* indices exceeded the WHO DEN and YF epidemic thresholds in all the villages (peri-urban: DF = 5–8; intra-urban: DF = 6–8). Such exceptionally elevated DEN and YF epidemic risks are often reported in major African urban centers such as Abidjan, Ouagadougou and Nairobi [5,29,39], but rarely in peri-urban areas, and thus should require particular attention. DEN and YF epidemic risks were similar among the peri-urban and intra-urban villages in terms of *Stegomyia* index categories (based on qualitative data), but differed quantitively for public health risks (*Ae. aegypti* densities and pupal indices based on quantitative methods were consistently higher in intra-urban than in peri-urban villages). This highlights the added value of combining the qualitative *Stegomyia* indices and quantitative pupal indices because *Stegomyia* indices alone are often poor predictors of arboviral outbreaks in Africa [48]. However, the peri-urban villages still had high-risk levels that are further compounded by additional sylvatic species, including *Ae. vittatus* that is a competent vector of DENV and YFV [35]. *Stegomyia* indices varied slightly across seasons, with DEN epidemic risks remaining permanently high and above the WHO epidemic thresholds year-round. YF epidemic risks were generally high across most seasons, except for moderate risks observed during LDS and SDS in the peri-urban villages, and during SDS in the intra-urban villages. This emphasizes the importance of year-round *Aedes* vector surveillance and control measures in all villages, as the risks do not significantly decrease during less productive periods. Such patterns of epidemic risks might be due to high container productivity driven by complex rainfall-water storage cycles. These social-climatic compensatory mechanisms could explain ongoing and recurrent DEN outbreaks, often coupled with YF cases, in Cocody-Bingerville.

### Implications for outbreak response and control policy

The present study provides valuable and timely insights into the potential eco-epidemiological role of local *Ae. aegypti* populations. Indeed, the ongoing DEN outbreak offers a unique opportunity to assess local *Aedes* vector ecology and DEN and YF risk dynamics under real-world conditions, allowing for more detailed and context-specific understanding of the disease epidemics. Our findings indicate that both peri-urban and intra-urban villages are exposed to consistently high epidemic risks. This underscores the need to expand *Aedes* vector surveillance and control to the peri-urban villages, which have historically received less attention. Indeed, failure to include peri-urban areas into outbreak responses may enable reservoir dynamics that sustain epidemics despite urban interventions. Furthermore, our study introduced, for the first time in Côte d’Ivoire, OPI, MEO and EDI indicators to evaluate *Ae. aegypti* oviposition activities. From our experience, this could enhance arboviral risk assessment as ovitrap-based surveillance is simpler in use, less resource-demanding, less time-consuming and cheaper than larval and pupal surveys [49,50]. Additionally, mass deployment of lethal ovitraps, sticky ovitraps or autocidal gravid ovitraps made of local discarded items could reduce *Ae. aegypti* numbers [51,52].

For maximal efficiency, focused LSM programs (container removals or larvidicing) targeting most productive containers during SDS identified as a strategic point for an efficient pre-emptive *Aedes* vector control in both peri-urban and intra-urban villages. LSM programs should be conducted in domestic ecozones in peri-urban villages, and in domestic and peridomestic ecozones in intra-urban villages. The national arbovirus control programs of Ministry of Health (MoH) and the local communities should be involved into and co-lead the interventions [53–55] to contain current and prevent future DEN outbreaks using optimal human, logistical and financial resources. For translational impact, we shared consolidated findings with key stakeholders (administrative and health authorities) and community members of Cocody-Bingerville by the awareness workshops held in Abidjan from 7–9 Octobre 2024. We engaged the workshop participants to adopt and apply our context-specific and eco-epidemiologically informed interventions described and to implement pilot programs targeting key larval habitats to reduce *Ae. aegypti* densities and arboviral risks in Cocody-Bingerville, including the peri-urban villages.

### Limitations and future research

Future research is required to gain a better understanding of DEN and YF outbreaks in the study areas. Indeed, due to resource limitations, we could not screen mosquito samples for DENV and YFV, and extrapolated adult densities via pupal indices. Our conclusion relies on one-year data only. Therefore, virological and serological studies can allow modeling human exposure and immunity and predicting future outbreak risks. Assessment of the biological, physicochemical and microclimatic characteristics of water-container systems and human behaviors will help to better understand aquatic habitat productivity and *Aedes* vector population dynamics. Future work should also explore *Aedes* adults’ abundance and behaviors (biting, resting cycles) and host preference and surveillance should last for at least two years to capture intra-annual and inter-annual variations for deeper understanding of local DENV and YFV transmission mechanisms.

### Conclusion

The study provided the first real-time data on *Aedes* population dynamics and DEN and YF epidemic risks in the peri-urban and intra-urban villages of Cocody-Bingerville during active outbreaks. Both village types harbor high numbers of *Ae. aegypti* immatures and larval habitats (tires, small and medium containers) that maintain epidemic risks above the WHO thresholds. *Ae. aegypti* densities were higher in LDS and lower in SDS in all villages, while pupal indices were lower in the peri-urban villages, but compensated by five additional *Aedes* species. Therefore, the peri-urban villages should be prioritized in *Aedes* vector surveillance and outbreak response plans. Ovitrap-based surveillance should be adopted for cost-effective monitoring, while integrated community-based LSM programs targeting identified containers could be applied in SDS for maximum efficiency. Analysis of DENV and YFV is suggested to model local transmission risks.

## Supporting information

S1 Fig Infestation of *Aedes aegypti* breeding sites in the peri-urban and intra-urban villages of Cocody-Bingerville, southeastern Côte d’Ivoire, from August 2023 to July 2024. A: Overall, B: Domestic ecozone, C: Peridomestic ecozone. Error bars indicate confidence intervals (95% CI). Others includes breeding containers made with brick holes, shoes, tarpaulins, wooden boxes, mortar, sheet metal, leaf armpits snail shells, underground puddles and tree holes.
(PDF)

S2 Fig *Aedes aegypti* container productivity in the peri-urban and intra-urban villages of Cocody-Bingerville, southeastern Côte d’Ivoire from August 2023 to July 2024. A: Overall, B: Domestic ecozone, C: Peridomestic ecozone. Error bars indicate confidence intervals (95% CI). Others includes breeding containers made with brick holes, shoes, tarpaulins, wooden boxes, mortar, sheet metal, leaf armpits snail shells, underground puddles and tree holes.
(PDF)

S1 Table Climate indicators recorded seasonally in the health district of Cocody-Bingerville, southeastern Côte d’Ivoire, from August 2023 to July 2024. (PDF)

S2 Table Species composition of mosquito adults emerged of eggs, larvae and pupae collected in the peri-urban and intra-urban villages of Cocody-Bingerville, southeastern Côte d’Ivoire, from August 2023 to July 2024. (PDF)

S3 Table Geographical distribution of *Aedes aegypti* in the peri-urban and intra-urban villages of Cocody-Bingerville, southeastern Côte d’Ivoire, from August 2023 to July 2024. (PDF)

S4 Table Seasonal variations in *Aedes aegypti*-positive breeding sites in the peri-urban and intra-urban villages of Cocody-Bingerville, southeastern Côte d’Ivoire, from August 2023 to July 2024. (PDF)

S5 Table Geographical distribution of *Aedes aegypti*-positive breeding sites in the peri-urban and intra-urban villages of Cocody-Bingerville, southeastern Côte d’Ivoire, from August 2023 to July 2024. (PDF)

S6 Table Geographical distribution in container productivity for *Aedes aegypti* pupae among peri-urban and intra-urban villages of Cocody-Bingerville, southeastern Côte d’Ivoire from August 2023 to July 2024. (PDF)

S7 Table *Aedes aegypti* oviposition indices in the peri-urban and intra-urban villages of Cocody-Bingerville, southeastern Côte d’Ivoire, from August 2023 to July 2024. (PDF)

S8 Table *Aedes aegypti* oviposition indices across ecozones in the peri-urban and intra-urban villages of Cocody-Bingerville, southeastern Côte d’Ivoire, from August 2023 to July 2024. (PDF)

S9 Table Correlations between *Stegomyia* indices, pupal indices and climate variables in the peri-urban and intra-urban villages of Cocody-Bingerville, southeastern Côte d’Ivoire, from August 2023 to July 2024. (PDF)
