# Supplementary figures and images for: Patterns of Aedes aegypti immature ecology and arboviral epidemic risks in peri-urban and intra-urban villages of Cocody-Bingerville, Côte d’Ivoire: Insights from a dengue outbreak
Source: PLoS One. 2026 Apr 30;21(4):e0324893. doi: 10.1371/journal.pone.0324893 (PMC13132252; doi:10.1371/journal.pone.0324893)

**A**

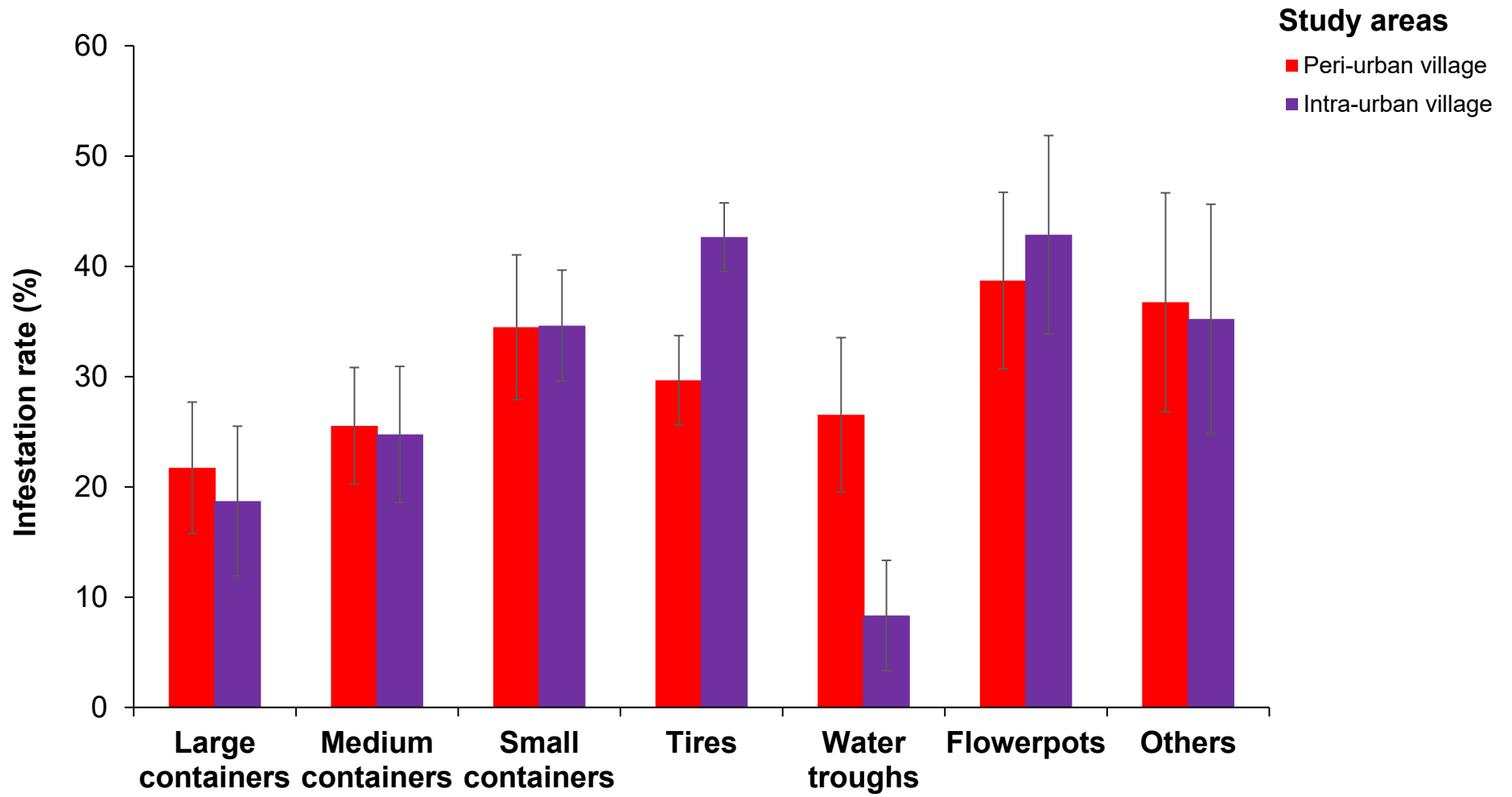

**B**

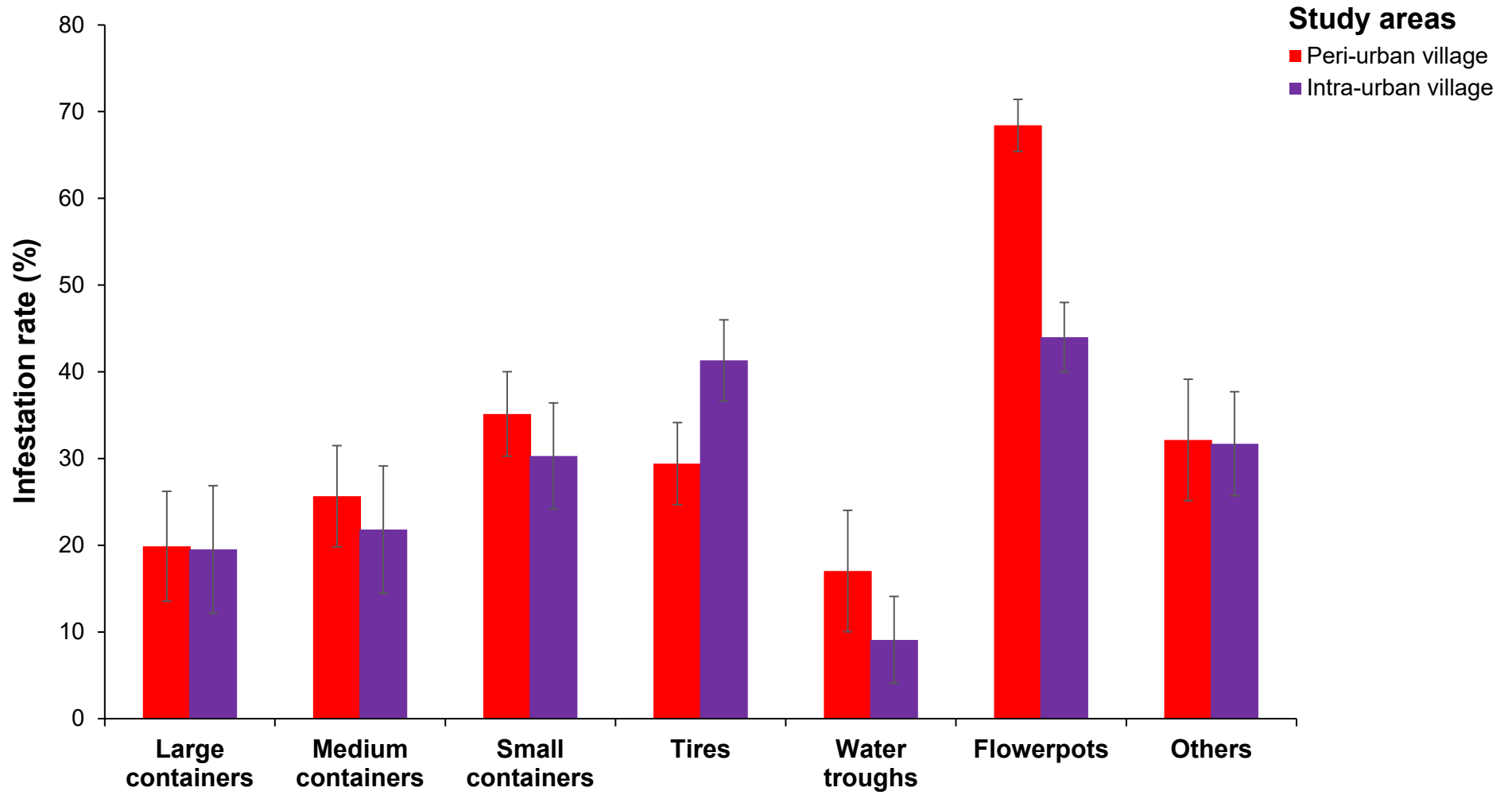

**C**

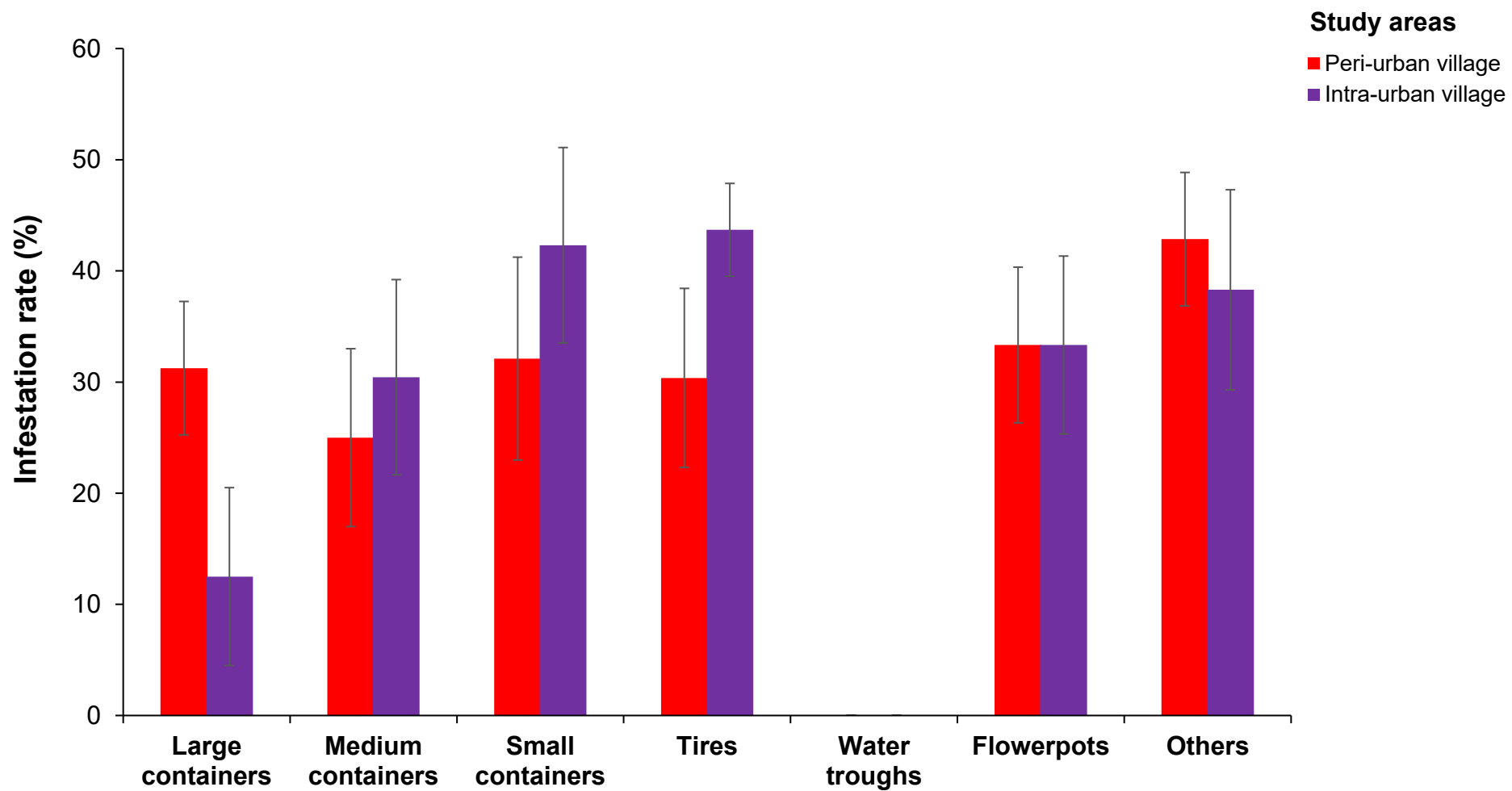

**S1 Fig**

Supplement: S1 Fig — A: Overall, B: Domestic ecozone, C: Peridomestic ecozone. Error bars indicate confidence intervals (95% CI). Others includes breeding containers made with brick holes, shoes, tarpaulins, wooden boxes, mortar, sheet metal, leaf armpits snail shells, underground puddles and tree holes. (PDF) [file pone.0324893.s001.pdf]

**A**

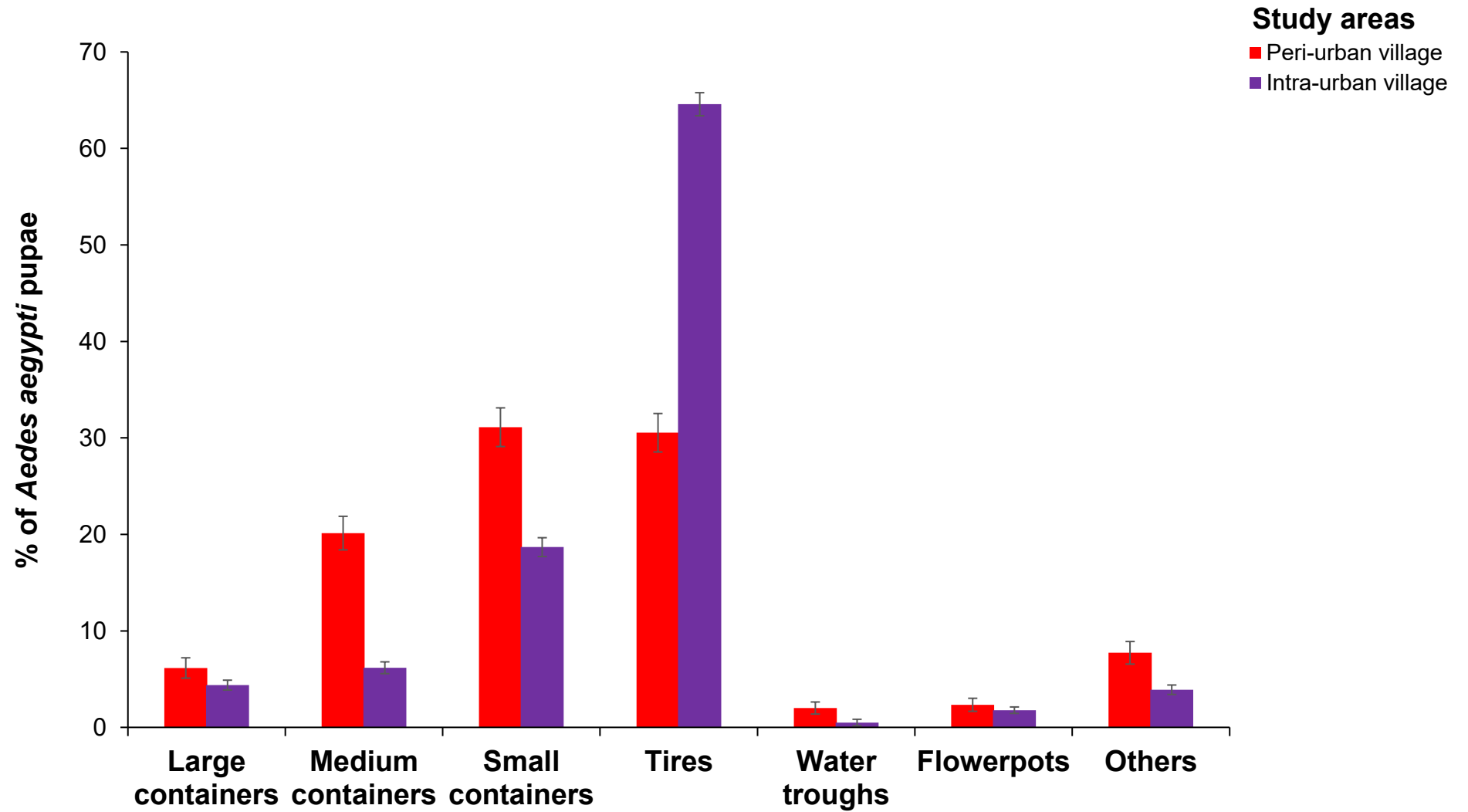

**B**

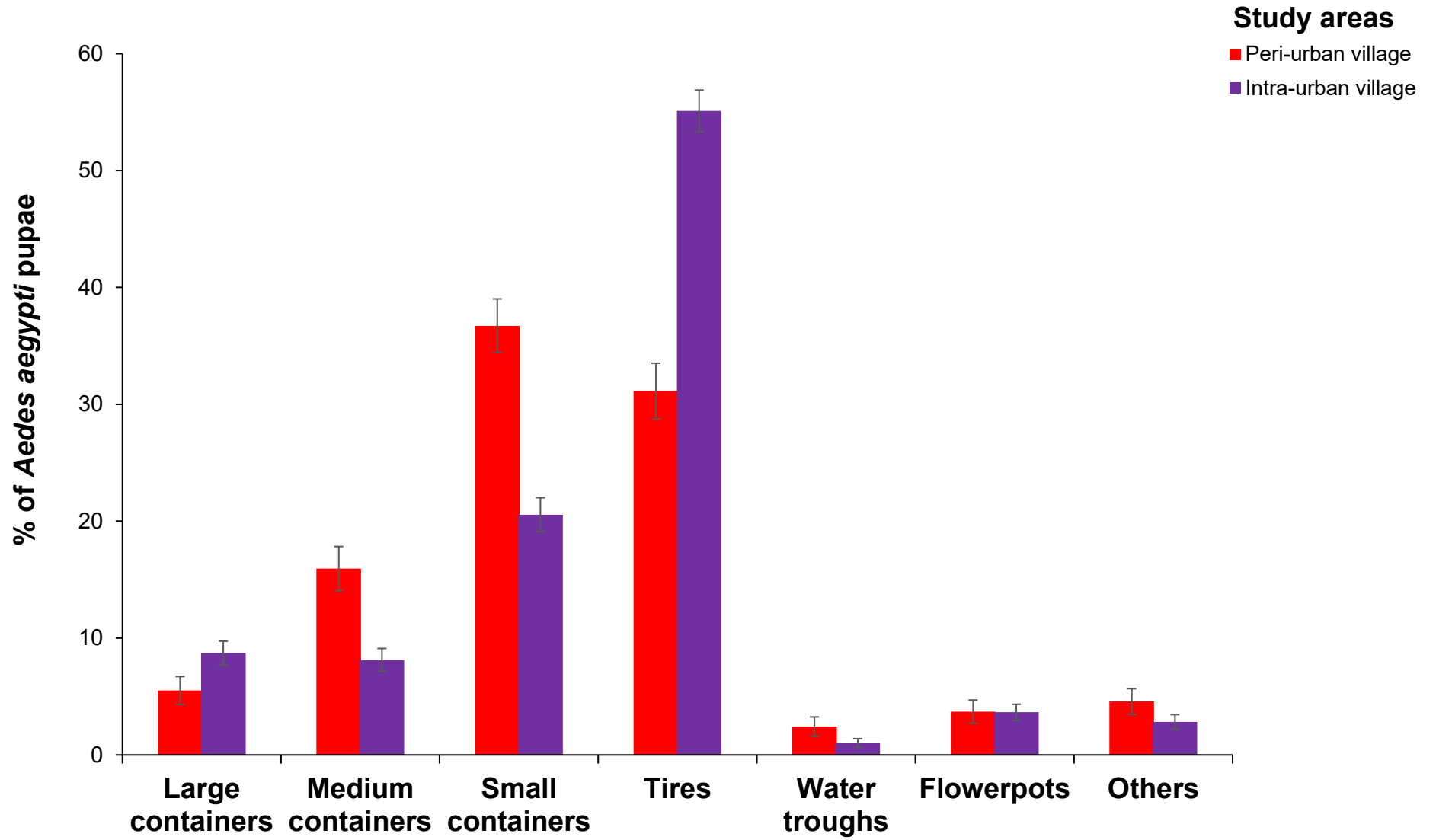

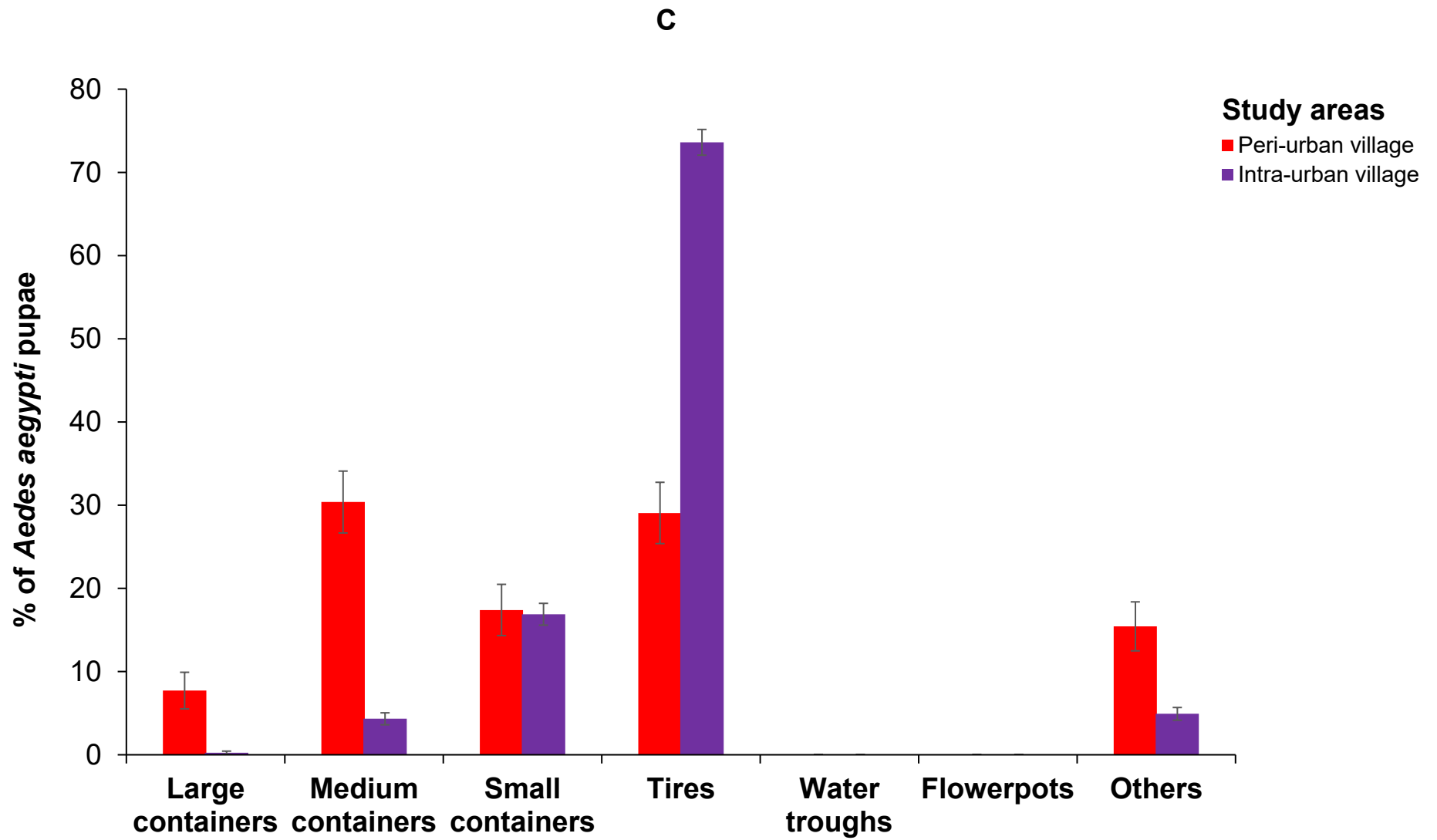

Supplement: S2 Fig — A: Overall, B: Domestic ecozone, C: Peridomestic ecozone. Error bars indicate confidence intervals (95% CI). Others includes breeding containers made with brick holes, shoes, tarpaulins, wooden boxes, mortar, sheet metal, leaf armpits snail shells, underground puddles and tree holes. (PDF) [file pone.0324893.s002.pdf]
