# Supplementary material for: Patterns of Aedes aegypti immature ecology and arboviral epidemic risks in peri-urban and intra-urban villages of Cocody-Bingerville, Côte d’Ivoire: Insights from a dengue outbreak
Source: PLoS One. 2026 Apr 30;21(4):e0324893. doi: 10.1371/journal.pone.0324893 (PMC13132252; doi:10.1371/journal.pone.0324893)
Supplement: S1 Table — (PDF) [file pone.0324893.s003.pdf]

**S1 Table. Climate indicators recorded seasonally in the heatlh district of Cocody-Bingerville, southeastern Côte d'Ivoire, from August 2023 to July 2024.**

| Season | Month | Rainfall (mm) | Temperature (°C) | Relative humidity (%) |
|---|---|---|---|---|
| Short dry season | August 2023* | 24.50 | 28.50 | 84.71 |
| | September 2023* | 9.60 | 23.51 | 65.68 |
| | **$Mean_1$ [95% CI]** | **17.05 [-77.6, 410]** | **26.01 [-5.70, 57.70]** | **75.19 [-45.70, 196.00]** |
| | **$Mean_2$ [95% CI]** | **17.05 [-77.6, 410]** | **26.01 [-5.70, 57.70]** | **75.19 [-45.70, 196.00]** |
| Short rainy season | October 2023* | 248.80 | 27.70 | 83.80 |
| | November 2023* | 130.30 | 28.40 | 81.70 |
| | **$Mean_1$ [95% CI]** | **189.55 [-563, 942,00]** | **28.05 [23.60, 32.50]** | **82,75 [69;40, 96.10]** |
| | **$Mean_2$ [95% CI]** | **189.55 [563.00, 942.00]** | **28.05 [23.60, 32.50]** | **82,75 [69.40, 96.10]** |
| Long dry season | December 2023* | 78.40 | 27.80 | 78.00 |
| | January 2024* | 26.70 | 34.04 | 78.81 |
| | February 2024 | 0.60 | 32.77 | 70.42 |
| | March 2024 | 99.40 | 34.89 | 75.68 |
| | **$Mean_1$ [95% CI]** | **52.55 [-276.00, 381]** | **30.92 [-8.72, 70.60]** | **78.40 [73.30, 83.60]** |
| | **$Mean_2$ [95% CI]** | **51.75 [-21.20, 124.00]** | **32.38 [27.30, 37.40]** | **75.73 [69.70, 81.70]** |
| Long rainy season | April 2024 | 141,10 | 33,51 | 74.97 |
| | May 2024 | 166.00 | 33.55 | 82.45 |
| | June 2024* | 361.70 | 26.50 | 84.90 |
| | July 2024* | 298.10 | 26.50 | 84.10 |
| | **$Mean_1$ [95% CI]** | **329.90 [-74.20, 734.00]** | **26.50 [26.50, 26,50]** | **84.50 [79,40, 89.60]** |
| | **$Mean_2$ [95% CI]** | **241.73 [73.70, 410.00]** | **30.01 [23.60, 36.50]** | **81.60 [74.40, 88.80]** |
| **Overall** | **$Mean_1$ [95% CI]** | **147.00 [32.20, 262.00]** | **27.90 [25.40, 30.30]** | **80.20 [74.80, 85.60]** |
| | **$Mean_2$ [95% CI]** | **132.10 [56.90, 207]** | **29.11[27.40,32.20]** | **78.82 [74.90, 82.70]** |

%: percentage, mm: millimeter, °C: Celsius degree, CI: confidence interval, *: month during which mosquitoes were collected, $Mean_1$: mean of climate variable during the period of mosquito collection. Mean $_2$: mean of climate variable during the climatic season,
