# Supplementary material for: Patterns of Aedes aegypti immature ecology and arboviral epidemic risks in peri-urban and intra-urban villages of Cocody-Bingerville, Côte d’Ivoire: Insights from a dengue outbreak
Source: PLoS One. 2026 Apr 30;21(4):e0324893. doi: 10.1371/journal.pone.0324893 (PMC13132252; doi:10.1371/journal.pone.0324893)
Supplement: S2 Table — (PDF) [file pone.0324893.s004.pdf]

S2 Table. Species composition of mosquito adults emerged of eggs, larvae and pupae collected in the peri-urban and intra-urban villages of Cocody-Bingerville, southeastern Côte d'Ivoire, from August 2023 to July 2024.

Village	Species	Eggs				Larvae				Pupae				Total			
		Female	Male	Total	%	Female	Male	Total	%	Female	Male	Total	%	Female	Male	Total	%
Peri-urban	*Aedes aegypti*	2768	2445	5213	92.97	2787	3061	5848	57.18	1032	1064	2096	41.69	6587	6570	13157	63.07
	Aedes dendrophilus	5	16	21	0.37	0	0	0	0.00	0	0	0	0	5	16	21	0.10
	Aedes fraseri	0	0	0	0	5	1	6	0.06	5	8	13	0.26	10	9	19	0.09
	Aedes lilii	0	0	0	0	8	12	20	0.20	0	0	0	0	8	12	20	0.10
	Aedes luteocephalus	0	0	0	0	2	13	15	0.15	0	0	0	0	2	13	15	0.07
	Aedes vittatus	1	0	1	0.02	70	76	146	1.43	24	16	40	0.80	95	92	187	0.90
	Anopheles gambiae	0	0	0	0	24	60	84	0.82	0	0	0	0	24	60	84	0.40
	Culex annulioris	0	0	0	0	621	882	1503	14.69	405	481	886	17.62	1026	1363	2389	11.45
	Culex nebulosus	126	149	275	4.90	755	918	1673	16.36	662	873	1535	30.54	1543	1940	3483	16.70
	Culex quinquefasciatus	0	6	6	0.11	287	235	522	5.10	51	62	113	2.25	338	303	641	3.07
	Eretmapodites chrysogaster	7	8	15	0.27	30	40	70	0.68	11	16	27	0.54	48	64	112	0.54
	Eretmapodites quinquevittatus	31	36	67	1.19	18	36	54	0.53	11	14	25	0.50	60	86	146	0.70
	Lutzia tigripes	3	6	9	0.16	77	207	284	2.78	110	182	292	5.81	190	395	585	2.80
	Toxorhynchites brevipalpis	0	0	0	0	1	2	3	0.03	0	0	0	0	1	2	3	0.01
	**Total**	**2941**	**2666**	**5607**	**100**	**4685**	**5543**	**10228**	**100**	**2311**	**2716**	**5027**	**100**	**9937**	**10925**	**20862**	**100**
Intra-urban	*Aedes aegypti*	4410	4862	9272	98.83	6935	6730	13665	87.18	2882	3349	6231	85.66	14227	14941	29168	90.22
	Aedes fraseri	0	0	0	0	21	6	27	0.17	1	2	3	0.04	22	8	30	0.09
	Aedes vittatus	0	1	1	0.01	0	0	0	0	8	25	33	0.45	8	26	34	0.11
	Anopheles gambiae	0	0	0	0	78	126	204	1.30	13	23	36	0.49	91	149	240	0.74
	Culex annulioris	0	0	0	0	484	431	915	5.84	224	303	527	7.24	708	734	1442	4.46
	Culex nebulosus	34	20	54	0.58	182	204	386	2.46	116	137	253	3.48	332	361	693	2.14
	Culex quinquefasciatus	14	22	36	0.38	111	157	268	1.71	47	86	133	1.83	172	265	437	1.35
	Eretmapodites chrysogaster	7	11	18	0.19	27	42	69	0.44	4	0	4	0.05	38	53	91	0.28
	Eretmapodites quinquevittatus	0	1	1	0.01	0	0	0	0	0	0	0	0	0	1	1	0
	Lutzia tigripes	0	0	0	0	40	101	141	0.90	25	29	54	0.74	65	130	195	0.60
	**Total**	**4465**	**4917**	**9382**	**100**	**7878**	**7797**	**15675**	**100**	**3320**	**3954**	**7274**	**100**	**15663**	**16668**	**32331**	**100**

% : percentage.
