# Supplementary material for: Patterns of Aedes aegypti immature ecology and arboviral epidemic risks in peri-urban and intra-urban villages of Cocody-Bingerville, Côte d’Ivoire: Insights from a dengue outbreak
Source: PLoS One. 2026 Apr 30;21(4):e0324893. doi: 10.1371/journal.pone.0324893 (PMC13132252; doi:10.1371/journal.pone.0324893)
Supplement: S3 Table — (PDF) [file pone.0324893.s005.pdf]

S3 Table. Geographical distribution of *Aedes aegypti* in the peri-urban and intra-urban villages of Cocody-Bingerville, southeaster Côte d'Ivoire, from August 2023 to July 2024.

Season	Ecozone	Peri-urban village		Intra-urban village		Total	
		n	%	n	%	n	%
Short dry season	Domestic	1066	8.10	1834	6.29	2900	6.85
	Peridomestic	348	2.64	1870	6.41	2218	5.24
	**Total**	**1414**	**10.75**	**3704**	**12.70**	**5118**	**12.09**
Short rainy season	Domestic	1357	10.31	2774	9.51	4131	9.76
	Peridomestic	999	7.59	2487	8.53	3486	8.24
	**Total**	**2356**	**17.91**	**5261**	**18.04**	**7617**	**18.00**
Long dry season	Domestic	2082	15.82	4603	15.78	6685	15.79
	Peridomestic	1260	9.58	4170	14.30	5430	12.83
	**Total**	**3342**	**25.40**	**8773**	**30.08**	**12115**	**28.62**
Long rainy season	Domestic	3644	27.70	5357	18.37	9001	21.27
	Peridomestic	2401	18.25	6073	20.82	8474	20.02
	**Total**	**6045**	**45.95**	**11430**	**39.19**	**17475**	**41.29**
Overall	Domestic	8149	61.94	14568	49.95	22717	53.67
	Peridomestic	5008	38.06	14600	50.05	19608	46.33
	**Total**	**13157**	**100**	**29168**	**100**	**42325**	**100**

n: number of *Aedes aegypti,* %: percentage.
