# Supplementary material for: Patterns of Aedes aegypti immature ecology and arboviral epidemic risks in peri-urban and intra-urban villages of Cocody-Bingerville, Côte d’Ivoire: Insights from a dengue outbreak
Source: PLoS One. 2026 Apr 30;21(4):e0324893. doi: 10.1371/journal.pone.0324893 (PMC13132252; doi:10.1371/journal.pone.0324893)
Supplement: S4 Table — (PDF) [file pone.0324893.s006.pdf]

S4 Table. Seasonal variations in *Aedes aegypti*-positive breeding sites in the peri-urban and intra-urban villages of Cocody-Bingerville, southeastern Côte d'Ivoire, from August 2023 to July 2024.

Village	Breeding site	Short dry season				Short rainy season				Long dry season				Long rainy season				Total			
		n_1	n_2	IR	PP	n_1	n_2	IR	PP	n_1	n_2	IR	PP	n_1	n_2	IR	PP	n_1	n_2	IR	PP
**Peri-urban**	Large containers	43	6	13.95	8.45	58	14	24.14	10.07	44	6	13.64	5.26	53	17	32.08	9.83	198	43	21.72	8.65
	Medium containers	38	7	18.42	9.86	92	29	31.52	20.86	58	10	17.24	8.77	90	25	27.78	14.45	278	71	25.54	14.29
	Small containers	74	21	28.38	29.58	107	34	31.78	24.46	154	59	38.31	51.75	161	57	35.40	32.95	496	171	34.48	34.41
	Tires	97	35	36.08	49.30	160	46	28.75	33.09	129	30	23.26	26.32	123	40	32.52	23.12	509	151	29.67	30.38
	Water troughs	18	2	11.11	2.82	12	3	25.00	2.16	1	1	100	0.88	18	7	38.89	4.05	49	13	26.53	2.62
	Flowerpots	0	0	0	na	5	3	60.00	2.16	11	4	36.36	3.51	15	5	33.33	2.89	31	12	38.71	2.41
	Others	5	0	na	na	29	10	34.48	7.19	13	4	30.77	3.51	51	22	43.14	12.72	98	36	36.73	7.24
	**Total**	**275**	**71**	**25.82**	**100**	**463**	**139**	**30.02**	**100**	**410**	**114**	**27.8**	**100**	**511**	**173**	**33.9**	**100**	**1659**	**497**	**29.96**	**100**
**Intra-urban**	Large containers	31	2	6.45	2.25	31	9	29.03	4.57	31	2	6.5	1.44	46	13	28.26	5.22	139	26	18.71	3.86
	Medium containers	37	9	24.32	10.11	58	17	29.31	8.63	54	9	16.7	6.47	53	15	28.30	6.02	202	50	24.75	7.42
	Small containers	49	11	22.45	12.36	80	29	36.25	14.72	94	30	31.9	21.58	138	55	39.86	22.09	361	125	34.63	18.55
	Tires	114	61	53.51	68.54	336	127	37.80	64.47	254	95	37.4	68.35	302	146	48.34	58.63	1006	429	42.64	63.65
	Water troughs	2	1	50.00	1.12	8	0	na	na	2	0	na	na	0	0	0	na	12	1	8.33	0.15
	Flowerpots	0	0	0	na	8	2	25.00	1.02	7	1	14.29	0.72	13	9	69.23	3.61	28	12	42.86	1.78
	Others	10	5	50.00	5.62	33	13	39.39	6.60	12	2	16.67	1.44	33	11	33.33	4.42	88	31	35.23	4.60
	**Total**	**243**	**89**	**36.63**	**100**	**554**	**197**	**35.6**	**100**	**454**	**139**	**30.6**	**100**	**585**	**249**	**42.6**	**100**	**1836**	**674**	**36.71**	**100**

n_1: number of wet containers, n_2: number of positive breeding sites, IR: infestation rate of positive breeding sites among wet containers, PP: proportion of each breeding site positive. The units of IR and PP are percentage (%), na: not applicable. Other is the category of breeding containers made up of brick holes, Shoes, Tarpaulins, wooden boxes, mortar, sheet metal, leaf armpits snail shells, underground puddles and tree holes.
