# Supplementary material for: Patterns of Aedes aegypti immature ecology and arboviral epidemic risks in peri-urban and intra-urban villages of Cocody-Bingerville, Côte d’Ivoire: Insights from a dengue outbreak
Source: PLoS One. 2026 Apr 30;21(4):e0324893. doi: 10.1371/journal.pone.0324893 (PMC13132252; doi:10.1371/journal.pone.0324893)
Supplement: S5 Table — (PDF) [file pone.0324893.s007.pdf]

S5 Table: Geographical distribution of *Aedes aegypti*-positive breeding sites in the peri-urban and intra-urban villages of Cocody-Bingerville, southeastern Côte d'Ivoire, from August 2023 to July 2024.

Villages	Breeding site	Domestic				Peridomestic				Total			
		N	n	IR (%)	PP (%)	N	n	IR (%)	PP (%)	N	n	IR (%)	PP (%)
**Peri-urban**	Larges containers	166	33	19,88	8,75	32	10	31,25	8,33	198	43	21,72	8,65
	Medium containers	230	59	25,65	15,65	48	12	25,00	10,00	278	71	25,54	14,29
	Small containers	387	136	35,14	36,07	109	35	32,11	29,17	496	171	34,48	34,41
	Tires	374	110	29,41	29,18	135	41	30,37	34,17	509	151	29,67	30,38
	Water troughs	47	13	27,66	3,45	2	0	0	0,00	49	13	26,53	2,62
	Flowerpots	19	8	42,11	2,12	12	4	33,33	3,33	31	12	38,71	2,41
	Others	56	18	32,14	4,77	42	18	42,86	15,00	98	36	36,73	7,24
	**Total**	**1279**	**377**	**29,48**	**100**	**380**	**120**	**31,6**	**100**	**1659**	**497**	**29,96**	**100**
**Intra-urban**	Larges containers	123	24	19,51	6,88	16	2	12,5	0,62	139	26	18,71	3,86
	Medium containers	133	29	21,80	8,31	69	21	30,4	6,46	202	50	24,75	7,42
	Small containers	231	70	30,30	20,06	130	55	42,3	16,92	361	125	34,63	18,55
	Tires	443	183	41,31	52,44	563	246	43,7	75,69	1006	429	42,64	63,65
	Water troughs	12	1	0	0	0	0	na	na	12	1	8,33	0,15
	Flowerpots	27	12	44,44	3,44	1	0	0,00	0,00	28	12	42,86	1,78
	Others	85	30	35,29	8,60	3	1	33,33	0,31	88	31	35,23	4,60
	**Total**	**1054**	**349**	**33,1**	**100**	**782**	**325**	**41,6**	**100**	**1836**	**674**	**36,71**	**100**

n_1: number of wet containers, n_2: number of positive breeding sites, IR: infestation rate of positive breeding sites among wet containers, PP: proportion of each breeding site positive. The units of IR and PP are percentage (%), na: not applicable. Other is the category of breeding containers made up of brick holes, Shoes, Tarpaulins, wooden boxes, mortar, sheet metal, leaf armpits snail shells, underground puddles and tree holes
