# Supplementary material for: Patterns of Aedes aegypti immature ecology and arboviral epidemic risks in peri-urban and intra-urban villages of Cocody-Bingerville, Côte d’Ivoire: Insights from a dengue outbreak
Source: PLoS One. 2026 Apr 30;21(4):e0324893. doi: 10.1371/journal.pone.0324893 (PMC13132252; doi:10.1371/journal.pone.0324893)
Supplement: S6 Table — (PDF) [file pone.0324893.s008.pdf]

S6 Table. Geographical distribution in container productivity for *Aedes aegypti* pupae among peri-urban and intra-urban villages of Cocody-Bingerville, southeastern Côte d'Ivoire from August 2023 to July 2024.

**Breeding site**	**Peri-urban**				**Intra-urban**				**Total**			
	**Domestic**		**Peridomestic**		**Domestic**		**Peridomestic**		**Domestic**		**Peridomestic**	
	**n**	**%**	**n**	**%**	**n**	**%**	**n**	**%**	**n**	**%**	**n**	**%**
Larges containers	82	63,57	47	36,43	265	97,07	8	2,93	347	86,32	55	13,68
Medium containers	237	56,16	185	43,84	247	64,16	138	35,84	484	59,98	323	40,02
Small containers	546	83,74	106	16,26	625	53,69	539	46,31	1171	64,48	645	35,52
Tires	463	72,34	177	27,66	1675	41,63	2349	58,37	2138	45,84	2526	54,16
Water troughs	36	100	0	na	31	100	0	Na	67	100	0	na
Flowerpots	55	100	0	na	111	100	0	na	166	100	0	na
Others	68	41,98	94	58,02	86	35,39	157	64,61	154	38,02	251	61,98
**Total**	**1487**	**70,94**	**609**	**29,06**	**3040**	**48,79**	**3191**	**51,21**	**4527**	**54,37**	**3800**	**45,63**

n: number of pupae, %: percentage of pupae, na: not applicable. Others are the category of breeding containers made up of brick holes, Shoes, tarpaulins, wooden boxes, mortar, sheet metal, leaf armpits, snail shells, underground puddles and tree holes.
