# Supplementary material for: Patterns of Aedes aegypti immature ecology and arboviral epidemic risks in peri-urban and intra-urban villages of Cocody-Bingerville, Côte d’Ivoire: Insights from a dengue outbreak
Source: PLoS One. 2026 Apr 30;21(4):e0324893. doi: 10.1371/journal.pone.0324893 (PMC13132252; doi:10.1371/journal.pone.0324893)
Supplement: S7 Table — (PDF) [file pone.0324893.s009.pdf]

S7 Table. ***Aedes aegypti*** **oviposition indices in peri-urban village and intra-urban villages of Cocody-Bingerville, southeastern Côte d'Ivoire, from August 2023 to July 2024.**

Village	Season	Ovitrap			OPI (%)		MEO (egg/ovitrap/week)		EDI (egg/ovitrap/week)	
		n_1	n_2	n_3	Mean	95% CI	Mean	95% CI	Mean	95% CI
**Peri-urban**	Short dry season	275	81	715	29.45	[45.13-57.48]	2.60	[1.81-3.39]	8.83	[6.67-11.00]
	Short rainy season	299	72	1061	24.08	[19.34-29.34]	3.55	[2.54-4.56]	14.74	[11.80-17.71]
	Long dry season	285	160	1878	56.14	[50.17-61.99]	6.59	[5.36-7.82]	11.74	[9.90-13.62]
	Long rainy season	265	136	1559	51.32	[45.13-57.48]	5.88	[4.48-7.19]	11.46	[9.31-13.60]
	**Total**	**1124**	**449**	**5213**	**39.95**	**[37.08-42.88]**	**4.64**	**[4.08-5.19]**	**11.61**	**[10.49-12.70]**
**Intra-urban**	Short dry season	273	137	2531	50.18	[44.21-56.15]	9.27	[7.61-10.92]	18.47	[15.99-20.96]
	Short rainy season	282	119	1777	42.20	[36.40-48.00]	6.30	[5.10-7.51]	14.93	[12.93-16.94]
	Long dry season	255	165	2194	64.71	[58.80-70.61]	8.60	[7.18-10.03]	13.30	[11.44-15.15]
	Long rainy season	259	146	2770	56.37	[50.29-62.45]	10.69	[8.78-12.60]	18.97	[16.26-21.71]
	**Total**	**1069**	**567**	**9272**	**53.04**	**[50.04-56.04]**	**8.67**	**[7.89-9.46]**	**16.35**	**[15.19-17.51]**

%: percentage, $n_{1:}$ number of ovitrap retrieved, n_2: number of positive ovitrap, n_3: number of eggs per ovitrap, OPI: oviposition positive index, MEO: means egg count per ovitrap, EDI: egg density index, CI: confidence interval.
