# Supplementary material for: Patterns of Aedes aegypti immature ecology and arboviral epidemic risks in peri-urban and intra-urban villages of Cocody-Bingerville, Côte d’Ivoire: Insights from a dengue outbreak
Source: PLoS One. 2026 Apr 30;21(4):e0324893. doi: 10.1371/journal.pone.0324893 (PMC13132252; doi:10.1371/journal.pone.0324893)
Supplement: S8 Table — (PDF) [file pone.0324893.s010.pdf]

S8 Table. ***Aedes aegypti*** **oviposition indices across ecozones in the peri-urban and intra-urban villages of Cocody-Bingerville, southeastern, Côte d'Ivoire from August 2023 to July 2024.**

Village	Season	Ecozone	Ovitrap			OPI (%)		MEO (egg/ovitrap/week)		EDI (egg/ovitrap/week)	
			n_1	n_2	n_3	Mean	95% CI	Mean	95% CI	Mean	95% CI
**Peri-urban**	Short dry season	Domestic	143	39	399	27.27	[30.18- 46.42]	2.79	[2.88- 6.78]	10.23	[8.19- 17.02]
		Peridomestic	132	42	316	31.82	[23.06- 39.06]	2.39	[1.99- 5.20]	7.52	[7.23- 15.94]
		**Total**	**275**	**81**	**715**	**29.45**	**[29.11- 40.48]**	**2.60**	**[2.97- 5.50]**	**8.83**	**[9.09- 15.25**
	Short rainy season	Domestic	149	32	458	21.48	[14.11- 27.22]	3.07	[1.89- 4.39]	14.31	[11.25- 19.07]
		Peridomestic	150	40	603	26.67	[16.49- 30.18]	4.02	[1.78- 4.63]	15.08	[8.97- 18.51]
		**Total**	**299**	**72**	**1061**	**24.08**	**[17.29- 26.71]**	**3.55**	**[2.22- 4.12]**	**14.74**	**11.36- 17.46]**
	Long dry season	Domestic	143	78	884	54.55	[53.66- 69.63]	6.18	[6.02- 9.58]	11.33	[10.26- 15.06]
		Peridomestic	142	82	994	57.75	[53.49- 70.03]	7.00	[6.35- 10.15]	12.12	[10.82- 15.88]
		**Total**	**285**	**160**	**1878**	**56.14**	**[55.99- 67.41]**	**6.59**	**[6.72- 9.31]**	**11.74**	**[11.27- 14.72]**
	Long rainy season	Domestic	134	60	663	44.78	[42.42- 59.1]	4.95	[4.59- 7.89]	11.05	[9.71- 14.68]
		Peridomestic	131	76	896	58.02	[52.60- 69.98]	6.84	[5.96- 9.88]	11.79	[10.29- 15.56]
		**Total**	**265**	**136**	**1559**	**51.32**	**[49.97- 62.28]**	**5.88**	**[5.79- 8.33]**	**11.46**	**[10.78- 14.39]**
	**Total**		**1124**	**449**	**5213**	**39,95**	**[40.13- 45.97]**	**4,64**	**[4.95- 6.16]**	**11,61**	**[11.80- 14.00]**
**Intra-urban**	Short dry season	Domestic	133	80	1427	60.15	[51.72- 68.58]	10.73	[8.14- 13.31]	17.84	[14.31- 21.37]
		Peridomestic	140	57	1104	40.71	[32.48- 48.95]	7.89	[5.78- 9.99]	19.37	[15.92- 22.82]
		**Total**	**273**	**137**	**2531**	**50.18**	**[44.21- 56.15]**	**9.27**	**[7.61- 10.92]**	**18.47**	**[15.99- 20.96]**
	Short rainy season	Domestic	143	68	1089	47.55	[39.27- 55.84]	7.62	[5.77- 9.46]	16.01	[13.29- 18.74]
		Peridomestic	139	51	688	36.69	[28.58- 44.80]	4.95	[3.41- 6.49]	13.49	[10.48- 16.50]
		**Total**	**282**	**119**	**1777**	**42.20**	**[36.40- 48.00]**	**6.30**	**[5.10- 7.51]**	**14.93**	**[12.93- 16.94]**
	Long dry season	Domestic	141	95	1267	67.38	[59.54- 75.21]	8.99	[6.88- 11.09]	13.34	[10.61- 16.06]
		Peridomestic	114	70	927	61.40	[52.33- 70.48]	8.13	[6.25- 10.02]	13.24	[10.85- 15.63]
		**Total**	**255**	**165**	**2194**	**64.71**	**[58.80- 70.61]**	**8.60**	**[7.18- 10.03]**	**13.30**	**[11.44- 15.15]**
	Long rainy season	Domestic	135	76	1363	56.30	[47.82- 64.77]	10.10	[7.79- 12.40]	17.93	[14.82- 21.05]
		Peridomestic	124	70	1407	56.45	[47.60- 65.30]	11.35	[8.19- 14.50]	20.10	[15.43- 24.77]
		**Total**	**259**	**146**	**2770**	**56.37**	**[50.29- 62.45]**	**10.69**	**[8.78- 12.61]**	**18.97**	**[16.26- 21.71]**
	**Total**		**1069**	**567**	**9272**	**53,04**	**[50.04- 56.04]**	**8.67**	**[7.89- 9.46]**	**16,35**	**[15.19- 17.51]**

%: percentage, n_1: number of ovitrap retrieved, n_2: number of positive ovitrap, n_3: number of eggs per ovitrap, OPI: oviposition positive index, MEO: mean egg count per ovitrap, EDI: egg density index, CI: confidence interval.
