# Supplementary material for: Patterns of Aedes aegypti immature ecology and arboviral epidemic risks in peri-urban and intra-urban villages of Cocody-Bingerville, Côte d’Ivoire: Insights from a dengue outbreak
Source: PLoS One. 2026 Apr 30;21(4):e0324893. doi: 10.1371/journal.pone.0324893 (PMC13132252; doi:10.1371/journal.pone.0324893)
Supplement: S9 Table — (PDF) [file pone.0324893.s011.pdf]

S9 Table. Correlations between *Stegomyia* indices, pupal indices and local climate variables in the peri-urban and intra-urban villages of Cocody-Bingerville, southeastern Côte d'Ivoire, from August 2023 to July 2024.

***Climate variable** / Aedes* index**	***Stegomyia* indices**						**Pupal indices**							
	**CI**		**HI**		**BI**		**PCI**		**PHI**		**PPI**		**HBI**	
	**Coefficient**	**p**	**Coefficient**	**p**	**Coefficient**	**p**	**Coefficient**	**p**	**Coefficient**	**p**	**Coefficient**	**p**	**Coefficient**	**p**
**Peri-urban village**														
Rainfall (mm)*	ρ = 0.4534	0.1388	ρ = 0.8408	< 0.0001*	ρ = 0.7570	0.0044*	ρ = 0.3671	0.2405	ρ = 0.5948	0.0414*	ρ = 0.6478	0.0228*	ρ = 0.5959	0.0409*
T (°C)*	ρ = -0.2807	0.3768	ρ = -0.7773	0.0029*	ρ = -0.5731	0.0514	ρ = -0.1727	0.5914	ρ = -0.3136	0.3209	ρ = -0.3455	0.2714	ρ = -0.3358	0.2858
RH (%)*	ρ = -0.1943	0.5451	ρ = 0.0216	0.9469	ρ = -0.1947	0.5443	ρ = -0.1943	0.5451	ρ = -0.3136	0.3209	ρ = -0.4318	0.161	ρ = - 0.3141	0.32
**CI**	na	na	ρ = 0.7368	0.0063*	ρ = 0.7762	0.0003*	ρ = 0.1295	0.6881	ρ = 0.1399	0.6646	ρ = 0.1399	0.6646	ρ = 0.6993	0.08290
**HI**	ρ = 0.7368	0.0063*	na	na	ρ = 0.9789	< 0.0001*	ρ = -0.0141	0.9654	ρ = 0.2035	0.5258	ρ = 0.1263	0.6957	ρ = 0.0947	0.7696
**BI**	ρ = 0.7762	0.0003*	ρ = 0.9789	< 0.0001*	na	na	ρ = -0.0841	0.7951	ρ = 0.1189	0.7128	ρ = 0.0419	0.8970	ρ = 0.0209	0.9484
**PCI**	ρ = 0.1295	0.6881	ρ = -0.0141	0.9654	ρ = -0.0841	0.7951	na	na	ρ = 0.9002	< 0.0001*	ρ = 0.9352	< 0.0001*	ρ = 0.9212	< 0.0001*
**PHI**	ρ = 0.1399	0.6646	ρ = 0.2035	0.5258	ρ = 0.1189	0.7128	ρ = 0.9002	< 0.0001*	na	na	ρ = 0.9790	< 0.0001*	ρ = 0.9860	< 0.0001*
**PPI**	ρ = 0.1399	0.6646	ρ = 0.1263	0.6957	ρ = 0.0419	0.8970	ρ = 0.9352	< 0.0001*	ρ = 0.9790	< 0.0001*	na	na	ρ = 0.9860	< 0.0001*
**HBI**	ρ = 0.6993	0.08290	ρ = 0.0947	0.7696	ρ = 0.0209	0.9484	ρ = 0.9212	< 0.0001*	ρ = 0.9860	< 0.0001*	ρ = 0.9860	< 0.0001*	na	na
**Intra-urban village**														
Rainfall (mm)*	ρ = 0.3023	0.3396	ρ = 0.7691	0.0034*	ρ = 0.7341	0.0066*	ρ = 0.1622	0.6145	ρ = 0.4966	0.1005	ρ = 0.5459	0.0663	ρ = 0.4318	0.161
T (°C)*	ρ = -0.3239	0.3044	ρ = -0.6067	0.0365*	ρ = -0.6262	0.0294*	ρ = 0	1	ρ = -0.2159	0.5003	ρ = -0.1943	0.5451	ρ = -0.1727	0.5914
RH (%)*	ρ = 0.0864	0.7896	ρ = -0.1408	0.6624	ρ = -0.1947	0.5443	ρ = -0.1406	0.663	ρ = -0.3239	0.3044	ρ = -0.2375	0.4573	ρ = -0.3023	0.3396
**CI**	na	na	ρ = 0.7368	0.0063*	ρ = 0.7762	0.0299*	ρ= 0.1296	0.6881	ρ= 0.1399	0.6646	ρ = 0.1399	0.6646	ρ = 0.0699	0.829
**HI**	ρ = 0.7368	0.0063*	na	na	ρ = 0.9790	< 0.0001*	ρ= -0.0141	0.9654	ρ = 0.2035	0.5258	ρ = 0.1263	0.6957	ρ = 0.0947	0.7696
**BI**	ρ = 0.7762	0.0299*	ρ = 0.9790	< 0.0001*	na	na	ρ= -0.0840	0.7951	ρ = 0.1189	0.7129	ρ = 0.0420	0.897	ρ = 0.0210	0.9484
**PCI**	ρ= 0.1296	0.6881	ρ= -0.0141	0.9654	ρ= - 0.0840	0.7951	na	na	ρ = 0.9002	< 0.0001*	ρ = 0.9352	< 0.0001*	ρ = 0.9212	< 0.0001*
**PHI**	ρ= 0.1399	0.6646	ρ = 0.2035	0.5258	ρ = 0.1189	0.7129	ρ = 0.9002	< 0.0001*	na	na	ρ = 0.9790	< 0.0001*	ρ = 0.9860	< 0.0001*
**PPI**	ρ = 0.1399	0.6646	ρ = 0.1263	0.6957	ρ = 0.0420	0.897	ρ = 0.9352	< 0.0001*	ρ = 0.9009	< 0.0001*	na	na	ρ = 0.9860	< 0.0001*
**HBI**	ρ = 0.0699	0.829	ρ = 0.0947	0.7696	ρ = 0.0210	0.9484	ρ = 0.9212	< 0.0001*	ρ = 0.9155	< 0.0001*	ρ = 0.9860	< 0.0001*	na	na

CI: container index, HI: house index, BI: Breteau index, PCI: pupae container index, PHI: pupae house index, PPI: pupae person index, HBI: human biting index, T: temperature, RH: relative humidity, %: percentage, mm: millimeter, °C: Celsius degree, na: not applicable, *: significant effects (p<0.05), -: negative correlation, p: p value. Results from Sperman (Shapiro-Wilk test was significant, p < 0.05) tests. *: climate variables.
